# Interactions of Catalytic Enzymes with n-Type Polymers for High-Performance Metabolite Sensors

**DOI:** 10.1021/acsami.2c20502

**Published:** 2023-02-07

**Authors:** David Ohayon, Dominik Renn, Shofarul Wustoni, Keying Guo, Victor Druet, Adel Hama, Xingxing Chen, Iuliana Petruta Maria, Saumya Singh, Sophie Griggs, Bob C. Schroeder, Magnus Rueping, Iain McCulloch, Sahika Inal

**Affiliations:** † Organic Bioelectronics Laboratory, Biological and Environmental Science and Engineering Division, King Abdullah University of Science and Technology (KAUST), Thuwal 23955-6900, Saudi Arabia; ‡ Catalysis Center, King Abdullah University of Science and Technology (KAUST), Thuwal 23955-6900, Saudi Arabia; § Physical Science and Engineering Division, KAUST, Thuwal 23955-6900, Saudi Arabia; ∥ Department of Chemistry, Chemistry Research Laboratory, University of Oxford, Oxford OX1 3TA, U.K.; ⊥ Department of Chemistry, University of College London, 20 Gordon Street, London WC1H 0AJ, U.K.

**Keywords:** organic bioelectronics, enzymatic sensors, glucose, catalytic enzymes, electron transporting (n-type) polymers, organic electrochemical transistor, conjugated polymers

## Abstract

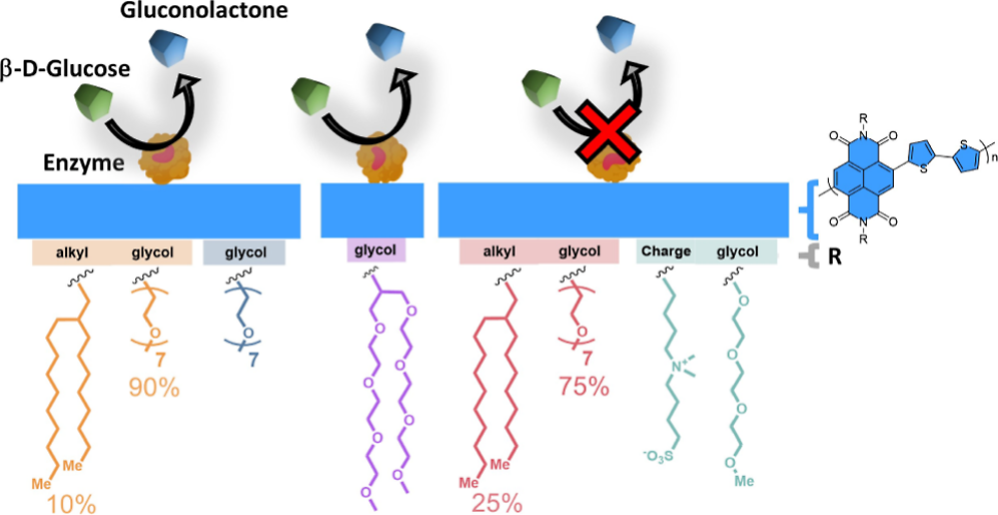

The tight regulation of the glucose concentration in the body is crucial for balanced physiological function. We developed an electrochemical transistor comprising an n-type conjugated polymer film in contact with a catalytic enzyme for sensitive and selective glucose detection in bodily fluids. Despite the promise of these sensors, the property of the polymer that led to such high performance has remained unknown, with charge transport being the only characteristic under focus. Here, we studied the impact of the polymer chemical structure on film surface properties and enzyme adsorption behavior using a combination of physiochemical characterization methods and correlated our findings with the resulting sensor performance. We developed five n-type polymers bearing the same backbone with side chains differing in polarity and charge. We found that the nature of the side chains modulated the film surface properties, dictating the extent of interactions between the enzyme and the polymer film. Quartz crystal microbalance with dissipation monitoring studies showed that hydrophobic surfaces retained more enzymes in a densely packed arrangement, while hydrophilic surfaces captured fewer enzymes in a flattened conformation. X-ray photoelectron spectroscopy analysis of the surfaces revealed strong interactions of the enzyme with the glycolated side chains of the polymers, which improved for linear side chains compared to those for branched ones. We probed the alterations in the enzyme structure upon adsorption using circular dichroism, which suggested protein denaturation on hydrophobic surfaces. Our study concludes that a negatively charged, smooth, and hydrophilic film surface provides the best environment for enzyme adsorption with desired mass and conformation, maximizing the sensor performance. This knowledge will guide synthetic work aiming to establish close interactions between proteins and electronic materials, which is crucial for developing high-performance enzymatic metabolite biosensors and biocatalytic charge-conversion devices.

## Introduction

Protein adsorption at solid–liquid interfaces is critical for many biological events, such as cell adsorption,^1^ transmembrane signaling,^2^ and the blood coagulation cascade.^3^ Protein adsorption is also one of the most challenging problems for biomedical device applications. For instance, thrombus formation on implants (and inflammatory responses)^3^ and hemodialysis membranes,^4^ biofouling of implanted devices and analytical chips intended for chronic use,^5^ and dental plaque formation^6^ are among the most common issues where protein adsorption leads to device failure. On the other hand, controlled protein adsorption is highly desirable for other devices and applications, such as biosensors and immunoassays,^7,8^ genome analysis,^9^ protein separation and purification,^10^ membrane filtration,^11^ and scaffold vascularization.^12^ Effective control over the protein adsorption process necessitates understanding the underlying mechanisms and the involved interactions with the surface. Unfortunately, no single universal understanding exists, and the current literature displays contention, reflecting the complexity of the adsorption phenomenon and protein–surface interactions.^13^ Various interfacial interactions (van der Waals, electrostatic, and hydrophobic) and surface physicochemical properties (charge, wettability, polarity, roughness, and morphology) and environmental conditions (protein bulk concentration, ionic strength, pH, and temperature) are thought to influence the adsorption behavior.^14,15^ A robust empirical model that links substrate surface properties to the resulting protein adsorption behavior would greatly benefit the design of new materials, particularly electronic materials for biosensors.

Electrochemical biosensors typically use proteins, such as enzymes or antibodies, as the biorecognition unit that binds the analyte.^16^ These proteins are often immobilized on the electrode or semiconductor surface. For the case of enzymatic metabolite sensors, the metabolite-binding and catalytic sites of the enzyme must be in a specific orientation with respect to the solution and the substrate, respectively, for efficient capture of the metabolite and transduction of the binding event. The enzyme immobilized on the surface should maintain its 3D solution structure as much as possible for efficient catalysis and target specificity. Therefore, the amount of protein bound, its orientation, and conformation are critical to sensor performance. Various strategies have been developed to immobilize enzymes on electronic surfaces. These include physical adsorption, covalent attachment, enzyme reconstitution, and protein engineering.^17,18^ The enzyme shelf life stability is often higher when attached to a surface compared to that of its soluble form.^19,20^ Among these methods, physical adsorption has been the most popular due to its simplicity. However, this approach lacks total control over the orientation of the protein, and denaturation or changes in the protein structure have been observed as a result of protein–surface interactions.^21^ It is thus essential to choose or decorate the electronic surface to allow for stable immobilization of the adsorbed enzyme with the desired orientation.

We have recently developed an electronic metabolite sensor where the enzyme was physically adsorbed on the organic semiconductor surface.^22−24^ The semiconductor was an electron-transporting (n-type) copolymer that was patterned on the channel and gate electrode of a microfabricated organic electrochemical transistor (OECT). For the case of the glucose sensor, glucose oxidase (GOx) was immobilized on the n-type polymer film. The device detected glucose (and lactate when lactate oxidase was used as the enzyme) in saliva with excellent sensitivity, selectivity, and a wide detection range of 6 orders of magnitude. The n-type polymer/GOx film also functioned as the anode of a glucose fuel cell, extracting enough power from biological media to drive small electronics.^22^ The sensor operation relied mainly on the O_2_ sensitivity of the n-type film, where its conductivity increased as the O_2_ amount decreased in the solution when glucose bound to GOx.^25^ The sensor results and spectroscopy analysis point to an intimate interface between the polymer film and the enzyme which allows the OECT to detect minute changes in O_2_ concentrations and, hence, enzyme activity at its surface. Despite the promise of this platform, it has not been clear why this particular polymer led to such high-performance sensors. Shedding light onto the enzyme adsorption process on this film would be very important to design new n-type semiconductors for biosensors, particularly when considering that the electronic performance of this polymer is behind that of the recently developed n-type materials.^26−28^

**Figure 1:**
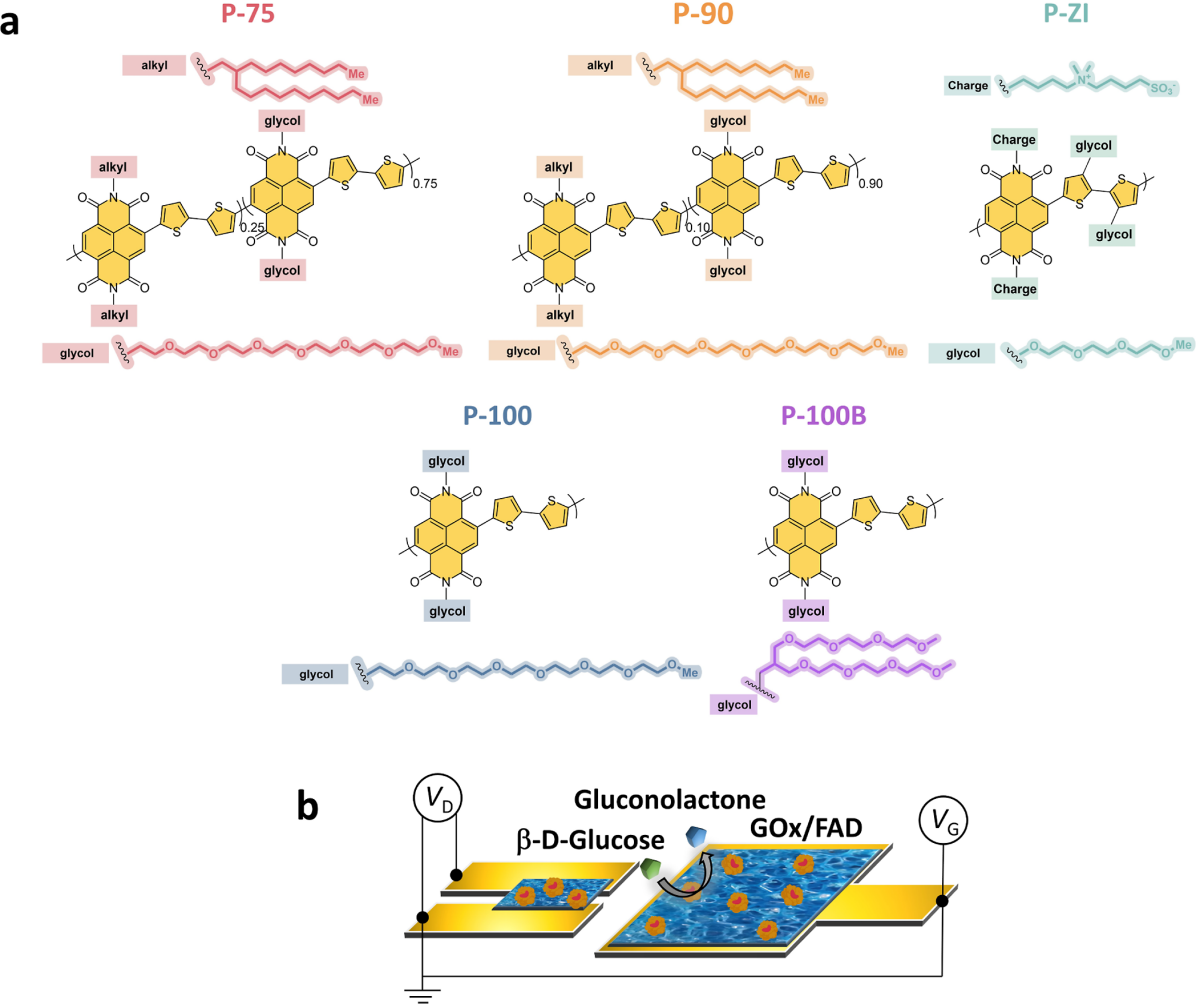
(a) Chemical structure of n-type polymers: the two copolymers with varying EG contents: P-75 and P-90, the fully glycolated (alkyl-free) analogue (P-100), P-100B with a branched EG side chain (branched analogue of P-100), and P-ZI with its zwitterions on the side chain. (b) OECT schematic highlighting the locations of the n-type film with adsorbed GOx (channel and gate). When glucose is present, it gets oxidized to gluconolactone by the active site (FAD) of GOx, following glucose + GOx (FAD) → gluconolactone + GOx (FADH_2_).

Here, we developed a series of n-type polymers based on the aforementioned polymer backbone (naphthalene diimide bi-thiophene, NDI-T2) but with various side chains (Figure 1a). All polymers had in common ethylene glycol (EG) side chains attached either to the NDI or the T2 unit, enabling solution processability and OECT operation in aqueous electrolytes.^29^ The first group comprises two copolymers where one monomer has a branched alkyl side chain, and the other contains a linear EG-based side chain (P-75 and P-90).^29^ We designed one homopolymer from the fully glycolated monomer (P-100)^29^ and another for which we replaced the linear EG chain with a branched one (P-100B).^30^ The last polymer of the series includes zwitterions on the side chain attached to the NDI unit (P-ZI). We first evaluated the glucose sensing performance of each film in the OECT configuration, as well as used a three-electrode setup to rule out any differences in the transistor performance of each polymer. We then characterized the film surface using water contact angle and ζ potential measurements, X-ray photoelectron spectroscopy (XPS), and atomic force microscopy (AFM). Using a quartz crystal microbalance with dissipation monitoring (QCM-D), we monitored the real-time enzyme adsorption behavior and quantified the amount of enzyme adhered to each polymer film. We calculated the resulting theoretical footprint of the enzyme and evaluated the viscoelastic properties of the adsorbed layer(s). We determined the structural composition of the protein layer on each film surface using circular dichroism (CD) and correlated the results with our GOx adsorption model. Our results show that although hydrophobic surfaces retain more enzymes, they also denature the enzyme to a higher degree than polar surfaces. On the hydrophilic/polar surfaces, GOx adsorbs loosely with a larger footprint, suggesting a flattened conformation. A hydrophilic, negatively charged, and smooth surface leads to the best-performing glucose sensors, indicating the need for a conformational change (flattening) to establish electronic communication with the semiconductor film. Focusing on semiconductor/enzyme interactions and the influence of side chains on enzyme adsorption (conformation) and sensor sensitivity, our work provides guidelines to design efficient bio-electronic hybrid materials with target application in enzyme-based electrochemical devices.

## Materials and Methods

### Materials

P-75, P-90, and P-100 were synthesized according to the previous protocols.^29^ The P-100B synthesis details can be found in a recent report (P-1G).^30^ P-ZI synthesis is described in the Supporting Information with NMR spectra provided in Figures S1–S4. d-Glucose, phosphate buffered saline (PBS), and GOx (*Aspergillus niger*, type X–S, lyophilized powder, 50 KU) were purchased from Sigma-Aldrich and used as received.

### Quartz Crystal Microbalance with Dissipation Monitoring

We conducted QCM-D measurements using a Q-sense analyzer (QE401, Biolin Scientific AB) with Cr/Au-coated quartz crystals before (used as a reference) and after coating with the polymer films. The changes in frequency (Δ*f*) and dissipation (Δ*D*) signals of the quartz-coated sensor were first measured in air (100 μL/min) and then in PBS, which was flown at a speed of 20 μL/min. After stabilizing the film in PBS (Δ*f* < 0.1 Hz per 5 min), we introduced GOx solution (10 mg/mL, PBS) in the chamber. The signals were recorded for 50 min, followed by a PBS rinsing step (10 min at 20 μL/min and then 200 μL/min for 1 h) to allow any loosely bound proteins to desorb. We measured the 3rd, 5th, 7th, 9th, and 11th harmonics and used the 7th one for analysis (see Supporting Information Discussion 2). The measured shifts in the frequency of the sensors were converted into changes in mass (Δ*m*) using the Sauerbrey equation
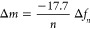
1where *n* is the number of the overtone selected for the mass quantification and −17.7 is a constant determined by the crystal’s resonant frequency, active area, density, and shear modulus. When examining surface events, such as protein adsorption, it is generally more desirable to focus on the higher harmonics of the quartz crystal. At lower harmonics, the trapping of the acoustic energy (assuming that the energy of the mechanical oscillations of the crystal is confined to its area) is relatively inefficient, and thus, the data are more likely to reflect bulk changes and external mechanical vibrations.^31^ We, therefore, chose to analyze the data using the seventh overtone, representing the best compromise between surface sensitivity and noise from our setup. We also fit the data using viscoelastic modeling (Kelvin–Voigt model, Q-Tools software, Biolin). Four harmonics (third, fifth, seventh, and ninth) were used for this model.

### Circular Dichroism

The GOx amino acid distribution and surface charge representation were obtained using the Expasy server and PyMOL Molecular Graphics System, Version 2.4.2, Schrödinger, LLC, respectively, by using the corresponding amino acid sequence (UniProtKB: P13006) for GOx for *A. niger*. PyMOL Molecular Graphics System, Version 2.4.2, Schrödinger, LLC was used to visualize the surface charges of GOx in its native state in solution.

CD spectra (190–270 nm) at 20 °C were recorded using a Jasco J-815 CD spectropolarimeter (Jasco Inc., Japan). A 1 mm-path-length quartz cell (Hellma GmbH) was used for solution samples. The GOx (UniProtKB: P13006) concentration in PBS was 1 mg/mL. Samples were prepared on quartz substrates by spin-coating the polymers using the same protocol as that mentioned above (see the Materials and Methods section). The enzyme was drop-casted on the polymer films and left to adsorb for 30 min. All spectra were recorded after accumulating 20 runs and smoothed using a fast Fourier-transform filter to minimize background effects. Quantitative prediction of the secondary structure was performed by deconvolution of the CD spectra using the CAPITO program.^32^ The program extracts the helical content at 220 nm, the β-strand at 206 nm, and the irregular structure at 199 nm only (neglecting the respective contributions of the other two elements at those wavelengths). Hence, the sum of the three secondary structural elements can differ from 100%. The represented spectrum corresponds to the average CD signal over triplicate experiments.

### Film Preparation

Polymer films were prepared on the corresponding substrates by spin-coating the polymers from a 4 mg/mL solution in chloroform (1000 rpm for 30 s, for P-75, P-90, P-100, and P-100B) or trifluoroethanol (1000 rpm for 30 s, for P-ZI).

### Water Contact Angle and Surface Free Energy Determination

The water contact angle of the polymer films was determined from static contact angle measurements using a KRUSS DSA100E drop-shape analyzer and Advance software (Germany).

### Atomic Force Microscopy

AFM measurements were performed using a Veeco Dimension 3100 scanning probe system. Samples were prepared on indium tin oxide substrates using the same conditions as those summarized above. The images of the films immersed in PBS were obtained using a Bruker ScanAsyst-fluid module mounted with ScanAsyst-fluid probes (nominal resonant frequency: 150 kHz, spring constant: 0.7 N/m). Gwyddion software was used for statistical data and post-treatment.

### X-ray Photoelectron Spectroscopy

XPS measurements were performed using a Kratos Axis Supra instrument equipped with a monochromatic Al Kα X-ray source (*h*ν = 1486.6 eV), which was operated at a power of 150 W and under ultra-high vacuum (in the range of 10^–9^ mbar). All spectra were recorded in the hybrid mode using electrostatic and magnetic lenses. The survey and high-resolution spectra were acquired at fixed S-5 analyzer pass energies of 80 and 20 eV, respectively. The obtained spectra were calibrated using the reference C 1s at 284.8 eV. We deconvoluted the spectra using XPSPeak4 software with Gaussian and Lorentzian methods and subtracted the background using the Tougaard method.

### OECT Fabrication, Characterization, and Operation of the Biosensor

The OECTs were microfabricated on glass substrates based on established protocols using standard photolithography and Parylene-C peel-off techniques.^33^ All polymers were spun at 1000 rpm for 30 s from a 4 mg/mL polymer solution (solvent: chloroform for P-75, P-90, P-100, and P-100B; trifluoroethanol for P-ZI). All devices had the same channel (width = 100 μm, length = 10 μm) and gate electrode (500 × 500 μm) dimensions. The OECT channel and gate surfaces were coated with the respective polymer. All channels and gates were incubated with a GOx solution (10 mg/mL in 1× PBS, pH 7.4) for 30 min at room temperature (20 °C). The sensing performance of the OECT biosensors was assessed via chronoamperometry using a Keithley 2602A source meter, where the drain voltage (*V*_D_) and gate voltage (*V*_G_) were fixed at 0.5 V. After a steady current baseline was obtained for the drain current (*I*_D_), we monitored the real-time changes in response to subsequent additions of increasing concentrations of glucose into the electrolyte. Solutions of the enzyme and glucose were stored at 4 °C. All electrical measurements were performed at room temperature. For all experiments, the electrolyte volume was kept at 40 μL. For an accurate comparison between devices, we normalized the device response to glucose
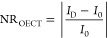
2where *I*_D_ and *I*_0_ are the current output at a given analyte concentration and zero analyte concentration, respectively.

We also measured the glucose sensitivity of Au electrodes (3 mm diameter) coated with the respective polymers in a three-electrode setup. Each electrode was coated with the corresponding polymer and functionalized with GOx according to the protocol detailed above. The polymer electrode was used as the working electrode, in combination with a Ag/AgCl reference electrode and platinum (Pt) coil counter electrode. We first evaluated the O_2_ sensitivity of our polymers by recording the electrode current in degassed and ambient atmospheres. The three-electrode setup was placed in a sealed glovebox flushed with N_2_ gas. The O_2_ content in the glovebox and electrolyte was monitored using an optical microsensor (PreSens Precision Sensing GmbH, Germany). The needle-type O_2_ microsensor (NTH-PSt7) has a spatial resolution down to 50 μm and a temporal resolution down to 3 s with a limit of detection of 0.03% O_2_. Once the O_2_ detection limit was reached, we performed chronoamperometry measurements in PBS-1× at −0.6 V versus Ag/AgCl using a PalmSens potentiostat. Once a steady current value was obtained, we stopped the N_2_ flush and opened the box to allow O_2_ to diffuse while recording the current changes. The experiment was stopped once a steady-state current value was obtained at an ambient O_2_ concentration. The polymer/GOx response to 1 mM glucose was then recorded under ambient conditions. We normalized the electrode’s current response to glucose with its response to O_2_
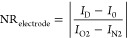
3*I*_D_ represents the stabilized current upon 1 mM glucose addition, and *I*_0_ is the baseline current under ambient conditions before the glucose addition.

### Safety Statement

No unexpected or unusually high safety hazards were encountered.

## Results and Discussion

### OECT Device Characteristics and Glucose Sensing Performance

**Figure 2:**
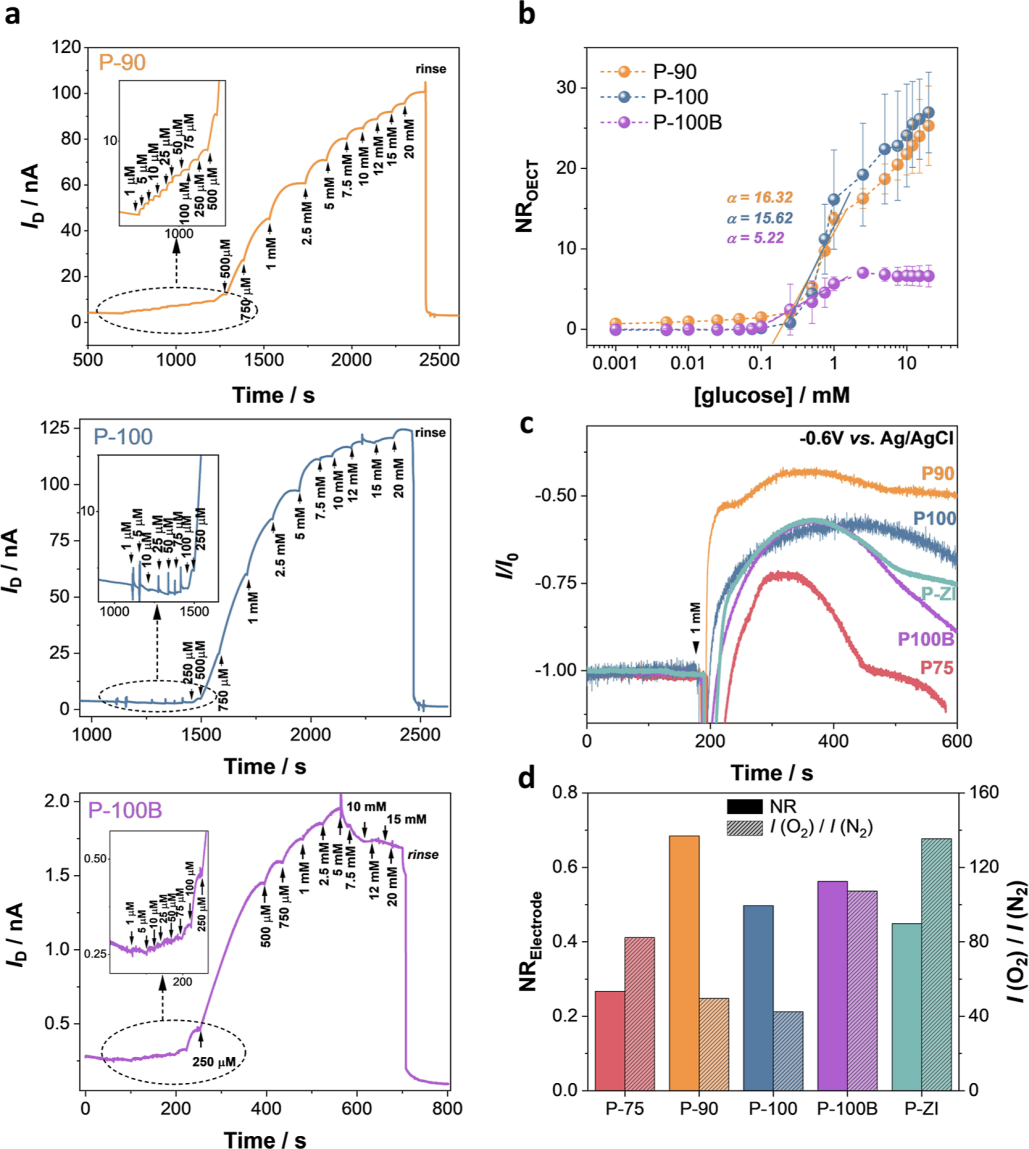
Performance of n-type OECTs and electrodes as glucose sensors. (a) Real-time response of the OECT (source-drain current, *I*_D_, as a function of time) as successive amounts of glucose are added to the electrolyte. The gate and drain voltages were kept constant at +0.5 V. The OECTs were operated using a planar gate configuration, where both the channel and gate were functionalized with GOx. Insets represent the real-time response of the devices to glucose concentrations lower than 250 μM. (b) Normalized response of the OECTs (NR_OECT_) to glucose. Error bars represent the standard deviation of at least three different devices. (c) Amperometric response of n-type conjugated polymer electrodes to 1 mM glucose. The working electrode was the n-type film functionalized with GOx, the reference electrode was Ag/AgCl, and the counter electrode was a Pt coil. The arrow represents the time point when 1 mM glucose was added into the electrolyte. (d) NR_electrode_ to 1 mM glucose (plain bars) and to O_2_ (patterned bars).

With Ag/AgCl as the gate electrode and PBS as the electrolyte, all devices showed enhancement mode OECT behavior (Figure S5). When using a planar Au electrode coated with the corresponding n-type polymer as the gate (as illustrated in Figure 1b), all channels displayed lower currents and transconductance (*g*_m_) values, while P-ZI could not be switched ON (Figure S6). We functionalized the active area of these devices with the enzyme. We evaluated the changes in the channel current (*I*_D_) as successive glucose concentrations were added to the measurement solution. As glucose was oxidized by GOx, the O_2_ amount in the electrolyte decreased as it was used for GOx regeneration. Since the n-type OECT current is inherently sensitive to changes in the O_2_ content of its environment, the GOx reaction with glucose led to an immediate increase in *I*_D_.^25^ Although all polymers are sensitive to O_2_ and all polymers except P-ZI operated sufficiently as an OECT, only P-90, P-100, and P-100B OECTs showed a current response to the oxidation of glucose (Figure 2a). Figure 2b compares the glucose sensitivity of these devices (NR_OECT_). P-90 and P-100 OECTs had similar sensing performance, while P-100B demonstrated the smallest response.

As *g*_m_ of each device differs (Figure S6), the OECT sensing performance may not be a direct indicator of enzyme/polymer interactions. To rule out the effect of the intrinsic OECT characteristics, we evaluated the glucose-sensing performances of our n-type polymers in a three-electrode configuration. However, the O_2_ sensitivity of each polymer may differ and hence their response to the same concentration of glucose. Therefore, first, we quantified the O_2_ sensitivity of our polymer-coated electrodes by measuring their respective reduction current in de-gassed and ambient atmospheres. All of our films showed an increase in their current when O_2_ was depleted from the electrolyte, with P-ZI and P-100B having the highest O_2_ sensitivity and P-90 and P-100 the lowest (Figure S7). We then measured the amperometric response of each polymer film to 1 mM glucose under ambient conditions (Figure 2c). All polymers, including P-75 and P-ZI which did not operate in OECTs, showed an increase in their current upon glucose oxidation. Figure 2d compares the normalized glucose- and the O_2_-triggered increases in the current of each electrode (see eq 3 in Materials and Methods). Although P-90 and P-100 are the least sensitive to O_2_, their current response to glucose is among the largest. Also, note that although P-100B is more sensitive to O_2_, the P100 and P100-B electrodes have comparable glucose sensing performance. On the other hand, the polymer with the highest O_2_ sensitivity, P-ZI, is not the most responsive to glucose. Given that these polymers sense glucose without relying on external mediators, their currents are not sensitive to hydrogen peroxide,^22,23^ and their response to glucose does not scale with their O_2_ sensitivity, we conclude that the sensor performance is largely governed by the differences in enzyme/polymer interactions.

### Surface Properties of n-Type Films

**Figure 3:**
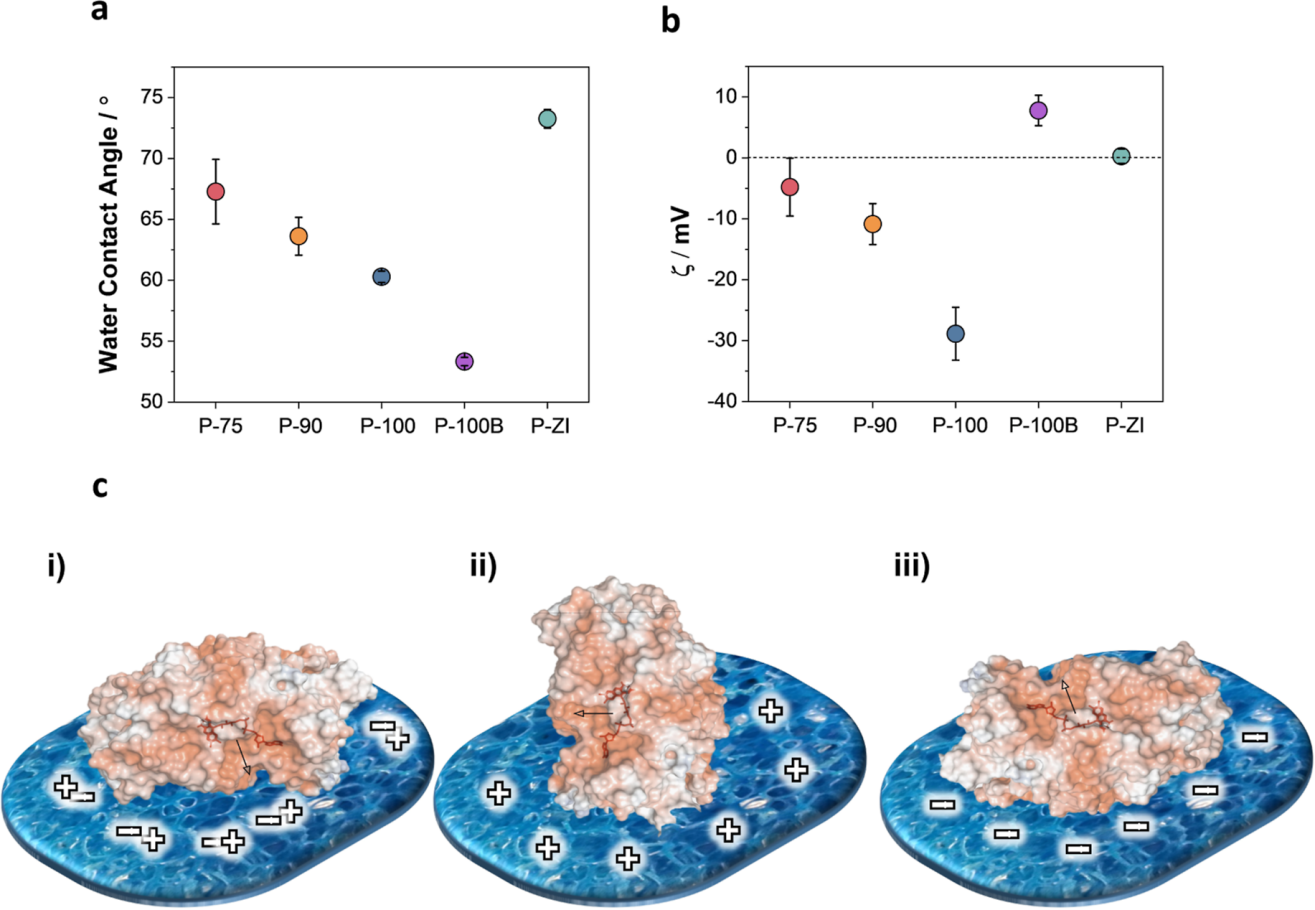
Surface properties of n-type films. (a) Water contact angle and (b) ζ potential of the n-type polymer films. Error bars represent the standard deviation of (a) nine and (b) four different measurements. (c) Orientations of native GOx from *A. niger* (PDB: 3QVP) as a function of surface charge: (i) “front-lying” orientation on a charge-neutral surface; (ii) “standing” orientation on a positively charged surface; and (iii) “back-lying” orientation on a negatively charged surface. Surface colors on GOx indicate positive and negative electrostatic potentials contoured from 50 kT/e (blue) to −50 kT/e (red). The cofactor is shown in stick representation and highlighted in red.

We sought to understand the origin of the differences in polymer/enzyme interactions by investigating first the film surface characteristics. Surface wettability and charge are thought to be the primary drivers of enzyme adsorption behavior (Supporting Information Discussion 1).^34^ We measured the water contact angle on the films as shown in Figure S8. Surface hydrophilicity increases with the EG content (from P-75 to P-100B), and P-ZI has the most hydrophobic surface (Figure 3a). Zeta potential (ζ) measurements revealed a net and increasingly negative surface charge when going from P-75 to P-90 and P-100, while P-100B and P-ZI had a positive and neutral surface, respectively (Figure 3b). Xie et al. showed that the surface charge influences the orientation of the adsorbed GOx; i.e., a positively charged surface leads to a preferred front-lying orientation, while a negatively charged surface is expected to induce a more favorable back-lying orientation.^35^ We used the PyMOL Molecular Graphics server to visualize the surface charges of GOx in its native state in solution (Figure S9) and estimated the possible orientation of GOx (i.e., assuming no conformational changes upon adsorption) on our films (Figure 3c). The front-lying orientation is expected to lead to a lower bioelectrocatalytic activity due to the hindered access to the substrate-binding site,^35^ which is the case for the P-ZI film. The most ideal enzyme orientation, where the enzyme active site is easily accessible and remains close to the electrode surface (back-lying orientation), is likely to occur on the negatively charged polymers, i.e., P-75, P-90, and P-100.

The surface roughness and morphology may affect the adsorption behavior of proteins, mainly if the lateral dimensions of the surface (nano)structures correspond to the length scale of the protein.^14,36,37^ We characterized the surface morphology of our films immersed in PBS using AFM. Although it is difficult to predict the effect of surface roughness alone on protein adsorption,^38^ studies showed that topological factors (e.g., roughness, feature size), combined with surface chemical variabilities, can influence protein adsorption characteristics.^36^ Empirically, the surface morphology became more fibrillary with the EG content, while the average surface roughness peaked for P-100B (7.3 nm), followed by P-90 (2.6 nm), P-100 (1.7 nm), and P-75 (1.5 nm). P-ZI displayed a morphology with small domains but no fibrillar content (Figure S11). Smaller-scale images (1 × 1 μm) exhibited features similar to those in larger scales (5 × 5 μm). The 3D representation of the morphologies highlighted differences in height and lateral dimensions of the nanostructures for each surface (Figure S11a). Except for P-100B, which displayed large “bumps” on the surface, all polymers had a “spikier” texture with a relatively homogeneous distribution, which was reflected in the average dimension distribution profiles of the surface (Figure S11b). We also analyzed the lateral surface dimensions by sampling each AFM image at three arbitrarily chosen regions (Figure S12). All polymers displayed much larger lateral dimensions (minimum 34.5 ± 3.6 nm) than the length scale of GOx (maximum 7.7 nm), suggesting that the surface morphology might not be the primary driver of enzyme adsorption and interactions with the polymers.

### Monitoring Enzyme Adsorption and Characterization of the Adsorbed Layer

**Figure 4:**
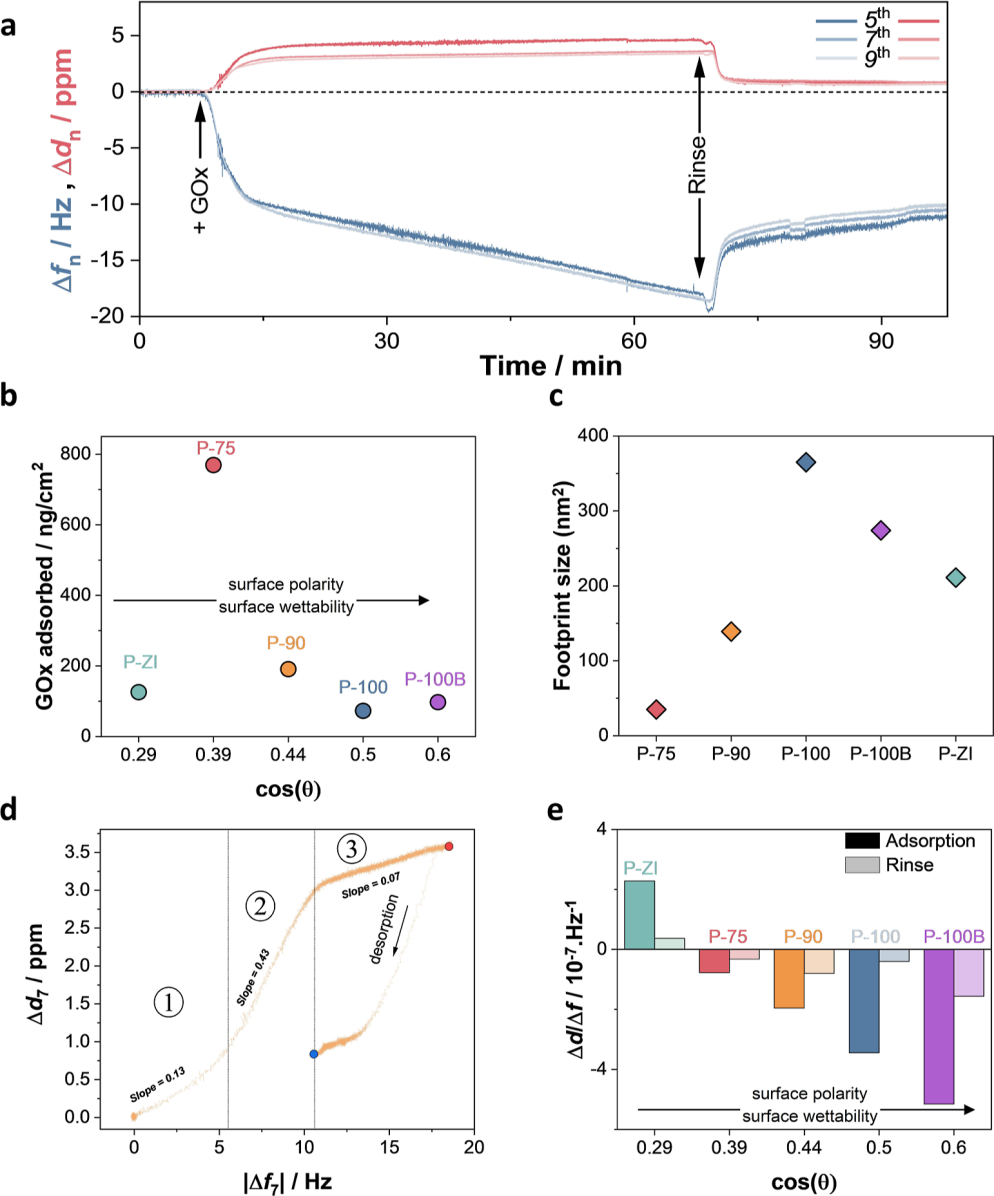
GOx adsorption on polymer films, analyzed using QCM-D. (a) P-90 QCM-D raw data reporting the change in frequency (Δ*f*_*n*_) and dissipation (Δ*d*_*n*_) for harmonics fifth, seventh, and ninth. (b) Corresponding mass taken up upon GOx adsorption on each film as a function of surface wettability [represented by cos(θ) where θ is the water contact angle]. The adsorbed mass per area was extracted from the data collected at the end of the rinsing process (see Figure S14). (c) Footprint of the enzyme on each polymer film. The footprint was calculated from the enzyme surface coverage and average dimensions of the crystal structure of a deglycosylated GOx molecule (60 Å × 52 Å × 77 Å),^40^ assuming an even distribution of the enzyme on a laterally homogeneous surface. (d) P-90 Δ*d* vs Δ*f* plots (seventh harmonic). The numbers define the linear regions in the plots with different slopes. The red and blue dots demark the end of the adsorption and desorption processes, respectively. (e) Δ*d*/Δ*f* ratio, calculated from the values measured at the end of the adsorption and the rinsing steps, as a function of wettability cos(θ).

We used QCM-D to monitor, in real time, the amount of enzyme adsorbing on polymer films and the viscoelastic properties of the protein layer. Figure 4a shows the changes in the frequency (Δ*f*) and dissipation (Δ*d*) signals from an exemplary P-90 film as GOx physically adsorbs on it. The same plots for other films are shown in Figure S13. The films were first left to swell in PBS. Once they were fully hydrated, QCM-D signals stabilized. The enzyme was then introduced into the chamber, causing a decrease in Δ*f* and an increase in Δ*d*, indicative of protein accumulation on polymer films and the softness of the formed layer. The enzyme solution was then replaced with PBS to wash away any unbound species, resulting in a physically adsorbed, stable protein layer. Proteins at the solid–liquid interface are generally considered non-rigid; thus, it is intuitive to analyze the QCM-D data using a viscoelastic model (Voigt) rather than the Sauerbrey equation.^39^ However, we could not consistently apply the Voigt model to all of our data (Figure S14) and determined the Sauerbrey model to be appropriate for our system. Figure 4b shows the mass increase on each sensor upon adsorption of GOx. We observed that the amount of hydrated enzyme remaining on the film depends on the polymer type. The P-75 film retained the highest GOx amount (769 ng cm^–2^), followed by P-90 (191 ng cm^–2^), P-ZI (126 ng cm^–2^), P-100B (97 ng cm^–2^), and P-100 (73 ng cm^–2^). P-75 has one of the most hydrophobic surfaces and a slight negative charge with a combination of alkyl and EG chains. Although P-ZI presents a similar hydrophobicity to P-75, the charge neutrality induced by its zwitterionic side chains seems to inhibit further mass uptake. As for negatively charged P-100 and positively charged P-100B, showing the most hydrophilic surfaces of the series, they do not attract more protein than P-90, where the protein amount is almost doubled compared to that on these surfaces. We conclude that protein absorption is more effective on hydrophobic surfaces populated with ideally a few negative charges. Alkyl side chains seem required for protein adsorption but should not dominate the surface. For example, when we tested a fully alkylated NDI-T2 analogue, namely, P-0, which has a much higher water contact angle (ca. 100°) than all the polymers studied,^29^ we saw that the enzyme adsorption did not improve further; in fact, it is limited to only 49 ng cm^2^ (Figure S15). Note that the mass values are calculated based on the assumption of an even distribution of the enzyme on a laterally homogeneous surface, and we do not consider the formation of clusters or multilayers.

From the adsorbed mass, we calculated the theoretical enzyme footprint on the films (Figure 4c). GOx had the largest footprint on P-0 (542 nm^2^, Figure S15c), where we hypothesized that the enzyme underwent significant conformational rearrangements due to the surface hydrophobicity (Supporting Information Discussion 1). The smallest enzyme footprint (35 nm^2^) was on the second top hydrophobic surface, i.e., P-75, suggesting a densely packed enzyme layer when considering its high amount therein. Among the rest of the glycolated films, the enzyme had the smallest footprint (139 nm^2^) on P-90, indicating either a more packed or slightly flattened enzyme layer. The case of P-100 is interesting. It has the lowest amount of enzyme with the largest footprint (after P-0), suggesting that the enzyme has an expanded conformation on this surface which we found to be highly negatively charged. Overall, these calculated footprints are generally larger than the theoretical footprint of GOx predicted from its crystal structure (21, 67, or 195 nm^2^)^37,41,42^ but are in line with other reported values on Au (between 528 and 823 nm^2^)^6^ and Au nanoparticles (between 250 and 300 nm^2^),^43^ suggesting that GOx flattens out to some degree during adsorption.^44,45^ Note that the native GOx dimensions are those of a deglycosylated GOx molecule, thus implying a larger structure for the protein in solution.^46^ We also stress the qualitative aspect of these calculations due to our assumptions. We conclude that the most hydrophobic surface without EG chains denatures the enzyme to the highest degree and that EG chains are required to minimize conformational changes. However, if EG chains fully dominate the surface, enzyme/polymer interactions are impaired, and the adsorbed proteins undergo partial denaturation. Note that we did not find a correlation between the amount adsorbed and the surface based on their charges (Figure 3a); however, the position of the binding site may still be necessary even after flattening of the enzyme on the surface, favoring the negatively charged surfaces.

Having determined the mass and the coverage area of the enzyme layer on each polymer surface, we next used the dissipation data to investigate the viscoelastic properties of the protein layer. The Δ*d* versus Δ*f* plots represent viscoelastic changes in the protein layer as the adsorption proceeds^31^ and contain a series of linear regions, each representing specific viscoelastic properties of the protein layer(s) (Figures 4d and S16, Supporting Information Discussion 2). The sign and the amplitude of these slopes indicate the rigidity of the formed layer, where a positive value implies the formation of a loose protein layer.^31^Figures 4d and S16 show that all polymers, except P-ZI, presented three linear regions during the adsorption process, suggesting similar kinetic behavior of the enzyme adsorption on these films. The first phase indicates a relatively rigid (small dissipation vs frequency) attachment until a critical surface coverage is reached. The second phase, characterized by a much steeper slope than that of phase 1, suggests the attachment of a viscoelastic stratum onto the first layer or loose (imperfectly coupled) binding of an additional rigid layer on the first. The last phase is associated with a further increase in the frequency, with a slight rise in dissipation, related to film thickening and possibly the removal of water molecules from the adlayers. For the glycolated polymer series, i.e., P-75, P-90, P-100, and P-100B, we observed a general increase in the slope values for all phases with the EG content (see Table S1). Some unbound protein detaches from the surfaces as the films are rinsed with PBS. We observe a different behavior of the enzyme on different films during the rinsing process. The enzyme layer on P-75 and P-90 displayed a constant dissipation while the frequency decreased, leading to a looser protein layer upon stabilization.

P-100 and P-100B exhibited an increase in frequency and dissipation (more evident for P-100B), possibly leading to a looser and/or more hydrated layer (Supporting Information Discussion 2). The increase in the dissipation for P-100 and P-100B can be due to a more expanded protein conformation which would increase the viscoelasticity of the bound molecules,^47^ implying that GOx adopts a more expanded conformation on these most hydrophilic surfaces. On the other hand, P-ZI displayed a very different adsorption behavior, presenting a two-phase process. The initial phase suggests the attachment of a very loose GOx layer, characterized by a high slope value (Table S1). In contrast, the second phase corresponding to the adsorption stabilization showed a negative slope value, indicating a stiffening of the enzyme layer as opposed to that on the glycolated surfaces. Furthermore, we observe a further rigidification of the enzyme layer upon rinsing, as illustrated by the negative dissipation value (Figure S16).

The final state of the enzyme layer(s) at the end of the adsorption and rinsing processes (red and blue dots in Figure 4d, respectively) is represented by the Δ*d*/Δ*f* ratio values. We examined the Δ*d*/Δ*f* ratios as a function of the polymer film surface wettability cos(θ), where θ is the water contact angle (Figure 4e).^48^ The Δ*d*/*Δf* ratio increased with wettability, suggesting a less rigid layer on a more polar/hydrophilic surface. Here, we note the unique case of P-ZI, showing a positive Δ*d*/Δ*f* ratio upon rinsing (due to a negative dissipation value with respect to the polymer baseline), presenting a significantly more rigid GOx layer than that on the glycolated surfaces. Furthermore, Figure 4b shows that the adsorbed GOx mass is generally inversely correlated with the surface wettability, where we found less dense layers, i.e., meaning a smaller amount of adsorbed protein, for more hydrophilic/loosely bound surfaces. We suggest that the hydrophobic character of P-75 and P-ZI led to an increased adsorbed mass due to stronger protein–protein interactions and denatured GOx. It has been postulated that in a denatured state, proteins tend to form more protein–surface and inter-protein interactions, leading to a more rigid layer, which correlates with the stiffer layer(s) we observed on P-75 and P-ZI.^48^

**Figure 5:**
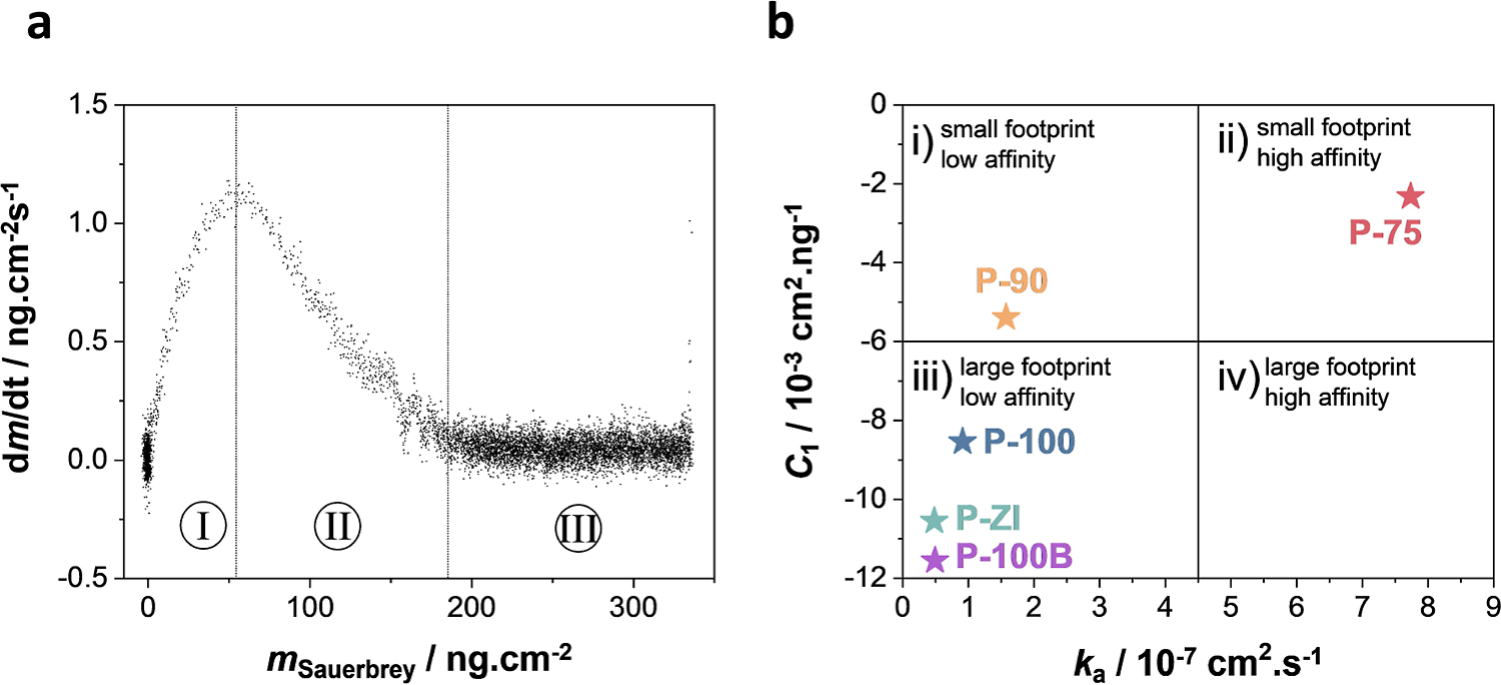
GOx adsorption kinetics. (a) d*m*/d*t* vs Sauerbrey mass uptake (seventh harmonic) plots for GOx adsorption on P-90 and (b) plot of *C*_1_ as a function of *k*_a_.

Next, we calculated the protein adsorption rate (d*m*/d*t*) and related it to the adsorbed mass (Figures 5 and S17). Figure 5a shows an exemplary plot of adsorption rate versus mass where we observed three main regions: (I) transport-limited regime, (II) reaction-limited regime, and (III) saturation regime.^39^ The linearly decreasing rate of the region (II) is characteristic of Langmuir-like adsorption, whereas its negative gradient is consistent with random sequential adsorption (RSA). Neither of the adsorption models seems to describe the behavior we observed. We, therefore, followed the suggestion of Nelson et al., who consider a mixture of immobile and mobile proteins with partially excluded space.^39^ We applied RSA model fitting (eq 4) for a qualitative understanding of the adsorption process:
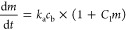
4where *k*_a_ is the rate constant (cm/s), *C*_1_ (cm^2^/ng) is a constant related to the steric factors of the adsorbed protein, and *c*_b_ is the protein concentration. Here, *k*_a_ concerns the rate constant of protein accumulation at low coverage (i.e., when protein–protein interactions are insignificant), whereas *C*_1_ reflects how the adsorbed protein molecules slow the subsequent adsorption of others. We found *C*_1_ to be linear to the surface area used by the enzyme at a given time during its adsorption (*A*_Ad_) (Figure S18a), while *k*_a_ was proportional to the adsorbed mass (Figure S18b); this indicates that the adsorption process is limited by the blocking effect of already adsorbed molecules. Figure 5b presents a plot of *C*_1_ versus *k*_a_, relating the adsorption kinetics to the footprint/affinity of the enzyme for the underlying surface.^39^ This plot is composed of four quadrants, each corresponding to different behavior. **Quadrant*i*** relates the adsorption of GOx on a surface to relatively low affinity. On such a surface, the protein footprint is expected to be relatively small. We found only P-90 in this quadrant, at the limit between **Quadrants*i*** and ***iii***, which correlates with the footprint calculated for P-90 in the range of other footprints reported on various gold surfaces. **Quadrant*ii*** corresponds to preservation of a small protein footprint on surfaces for which it has high affinity. As postulated by Nelson et al., this is not an apparent behavior; however, it correlates with the calculated footprint and our hypothesis that GOx aggregates in the form of clusters or multilayers on P-75 due to strong protein–protein interactions. **Quadrant*iii*** represents protein adsorption with a large footprint on surfaces for which it has a low affinity, and P-100, P-100B, and P-ZI are in this quadrant. Finally, **Quadrant*iv*** illustrates a surface for which GOx would have a high affinity, adsorbing with a large footprint.

Note that except for P-ZI, we observed a trend where the steric parameter *C*_1_ increased with the surface wettability, as illustrated in Figure S19. The low values of *C*_1_ for less polar surfaces have been suggested to indicate a higher packing density of proteins.^48^ Our results for P-75 are in agreement with this hypothesis. QCM-D measurements showed that the highest amount of GOx was adsorbed by the P-75 film, presenting the lowest *C*_1_ value, which could be due to the aggregation of the proteins in the form of clusters that leave a free surface for incoming proteins to adsorb on.^48^ Furthermore, except for P-ZI, the adsorption rate constant *k*_a_ decreased with surface hydrophilicity (Figure S19), suggesting slower adsorption kinetics on more polar surfaces, attributed to the lower enzyme affinity and possible flattening of the protein.

### Characterization of Enzyme-Adsorbed n-Type Films

**Figure 6:**
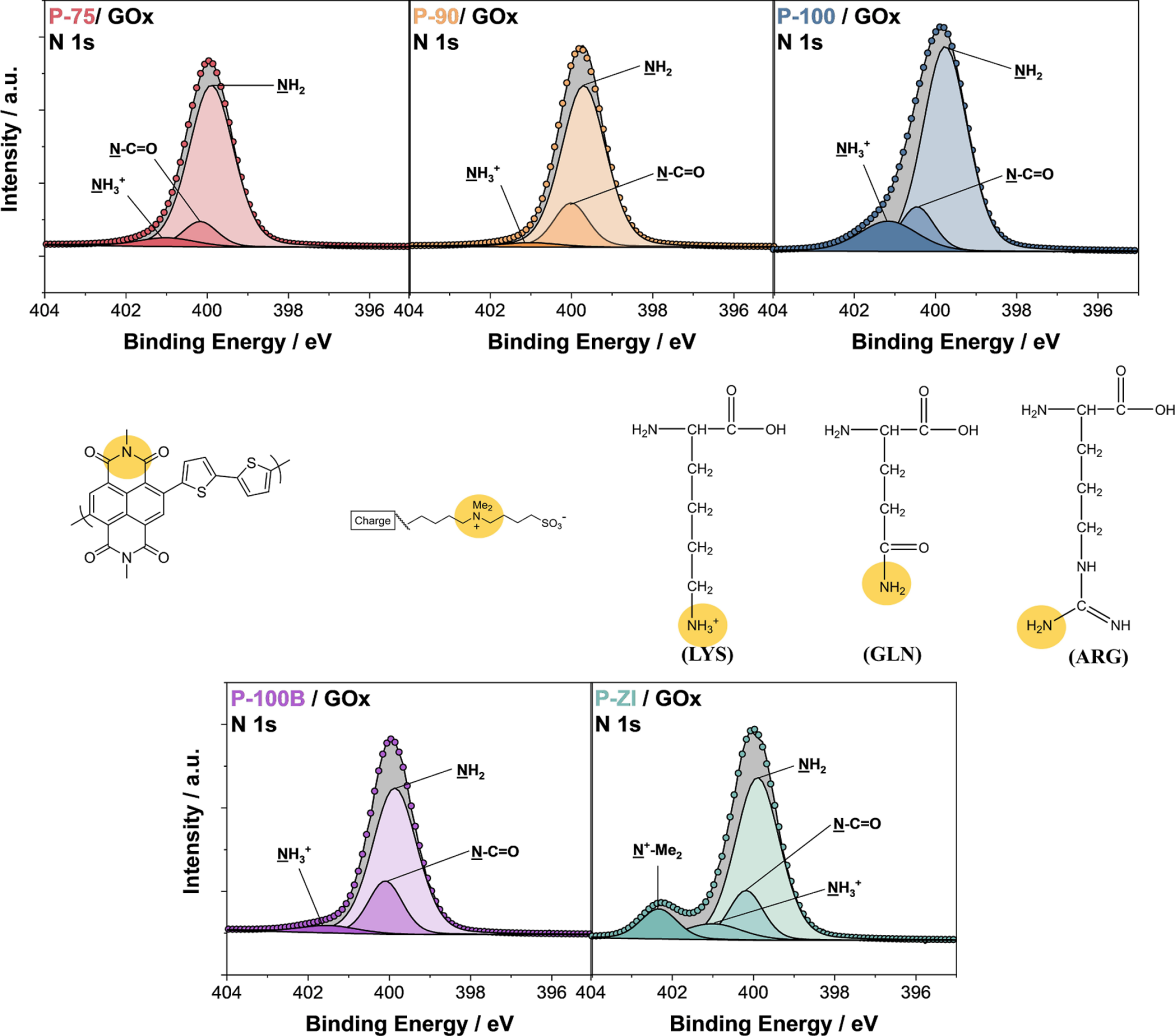
High-resolution N 1s XPS spectra of the polymer films after enzyme adsorption. Yellow circles highlight the chemical bonds involving N atoms in the polymers, including the NDI backbone and side chains and those in the primary amino acids present in GOx. The amino acids displayed in the figure correspond to a selected few in the protein sequence: lysine (LYS), glutamine (GLN), and arginine (ARG).

Since all the techniques that we used above are somehow indirect, we used XPS analysis to evidence the enzyme layer on the surface and identify which surface groups interacted with the protein. We recorded the XPS N 1s spectra shown in Figure 6 (Table S2) because GOx comprises various amino acids that contain nitrogen atoms (see the list of amino acids with their properties and fraction in the structure in Table S3).^49^ On the other hand, all polymers have only one kind of nitrogen bond from the NDI moiety (N–C=O). Only P-ZI involves an additional nitrogen bond, −NMe_2_^+^, arising from its zwitterionic side chain. We compared the relative area of the NH_2_ peak to the total area of the N 1s region for each polymer to estimate which film contained more enzymes on its surface (Table 1). We observed that P-75 had the highest contribution from the −NH_2_ peak (attributed to various amino acids). This analysis confirmed that the zwitterionic polymer repelled to some extent the enzyme. XPS N 1s analysis suggests that increasing the amount of the EG content in the side chains decreased the amount of enzyme adsorbed. All these results are in agreement with our QCM-D results.

**Table 1:** Relative Contribution of the NH_2_ Area (*A*_NH2_) to the N 1s Region (*A*_TOT_)

polymers	*A*_NH2_/*A*_TOT_
P-75	0.840
P-90	0.805
P-100	0.736
P-100B	0.744
P-ZI	0.661

We also investigated the C 1s spectra of the polymers before and after enzyme adsorption (Figures S20 and S21, respectively, Tables S4–S6 for the bonds). We could detect C–O bond signals from pristine films, suggesting that some EG chains are located on the uppermost surface. We observed an increase in the relative contribution of this bond with EG substitution or branching (Table S5), in agreement with previous reports.^50^ Once the enzyme was added to the films, only P-100 displayed two additional peaks in the deconvoluted C 1s spectra (Figure S21 and Table S6), attributed to C=C–N (285.77 eV) and C–OOH (288.498 eV) from GOx amino acids.^51^ S 2p XPS shows similar additional features only for this film (Figure S22 and Table S7). Considering that the enzyme mass on this film is not the highest in the series, these additional signals suggest an expanded/flattened conformation of the enzyme on the P-100 surface, exposing these amino acids.

Moreover, we observed a significant shift in the C=C bond in P-90 (−0.115 eV), P-100 (−0.081 eV), and P-100B (−0.028 eV), to lower binding energies, compared to that in P-75 (+0.024 eV) and P-ZI (+0.092 eV) which had instead shifted to higher binding energies (Table S8). These shifts indicate an increased electron density around the C=C bonds of P-90, P-100, and P-100B. P-90 and P-100 displayed the most drastic shift (>0.2 eV) of the C=O bonds of the NDI moieties to lower binding energies (Table S8). Previously, we found similar interactions of the enzyme GOx with the C=O and C=N peaks of the P-90 polymer using in situ Raman spectroscopy.^22^ We also noticed important shifts in the C–O bonds in most polymers to higher binding energies, indicating interactions of GOx with the EG side chains of these polymers.^52^ We found the largest shifts for P-100 (+0.409 eV) and P-90 (+0.135 eV), followed by P-ZI (+0.125 eV), P-75 (+0.123 eV), and lastly, P-100B (+0.063 eV). It is interesting to note that the shift of C–O is much larger for P-100 (+0.409 eV) compared to that for P-100B (+0.063 eV), implying stronger interactions of GOx with P-100 than with P-100B, in line with the higher sensitivity of the P-100-based glucose sensor. Branching makes the surface more hydrophilic and positively charged, which seems to hinder interactions with the enzyme and the position of the reaction center (Supporting Information Discussion 1).

Next, we used CD to investigate the secondary structure of the enzyme upon adsorption on different polymers. Proteins tend to lose the α-helical content and gain the β-sheets and random coil/irregular content upon adsorption on surfaces.^15,53^ CD studies of GOx adsorbed or entrapped on various substrates, such as the Au nanoparticles,^43^ Nafion matrix,^54^ and self-assembled monolayers,^37,45,55^ revealed a characteristic increase in the β-sheet content with a decrease in the α-helical content upon biohybrid formation. The α-helical content of GOx is closely linked to its activity. Glucose biosensors with GOx adsorbed in an α-helix conformation exhibited a much higher sensitivity to glucose than those with GOx adsorbed in a β-sheet conformation.^45^ Thus, the α/β ratio is a good indication of the enzyme conformational change and resulting activity. The CD spectra that we collected for each polymer film/GOx complex are shown in Figure S23. We deconvoluted the raw CD spectra using the CAPITO program as a qualitative tool to compare the conformation adopted by GOx upon adsorption on our polymers.^32^Table 2 represents the assessment of the GOx secondary structure upon adsorption on each polymer film after CD spectrum deconvolution. Overall, the best-performing polymers in OECT sensors, P-90 and P-100, display a more conserved content of secondary structure elements of GOx, showing similar α/β ratios and the least decrease in the α content. A further loss in the helical content is observed for the more polar and hydrophilic P-100B. Together with the decrease in the α helix, the β sheet content increased more significantly for the more hydrophilic P-100 and P-100B compared to that for P-90. These results suggest a more flattened conformation on these surfaces compared to that on P-90, in agreement with our QCM-D and XPS results. Surprisingly, the CD data indicate changes in the GOx structure upon adsorption on P-75 similar to P-100B despite the surfaces presenting opposite charges and different hydrophilicity. Recall that we could not observe any response to the glucose with the P-75 OECT sensor, and the polymer showed the least amperometric response to glucose despite being the one with the highest oxygen sensitivity and the highest amount of GOx. A possible reason is the unfavorable enzyme orientation on P-75 due to the lower negative charge and higher hydrophobicity. The P-ZI film, on the other hand, exhibited significant changes with a complete loss of the α-helical content and an increase in the irregular structure, consistent with our hypothesis of significant unfolding/denaturation on this film.

**Table 2:** Relative Content of Secondary Structural Elements of GOx Protein upon Adsorption on n-Type Polymers^a^

	α-helix	β-sheet	irregular	α/β
GOx (native)	0.09	0.48	0.52	0.19
P-75	0.01	0.53	0.53	0.02
P-90	0.02	0.51	0.53	0.04
P-100	0.02	0.55	0.51	0.04
P-100B	0.01	0.54	0.52	0.02
P-ZI	0.00	0.52	0.56	0.00

a The helical, β-strand, and irregular contents were calculated individually, and therefore, the sum of the three secondary structural elements may not be equal to 100%.

## Discussion

**Figure 7:**
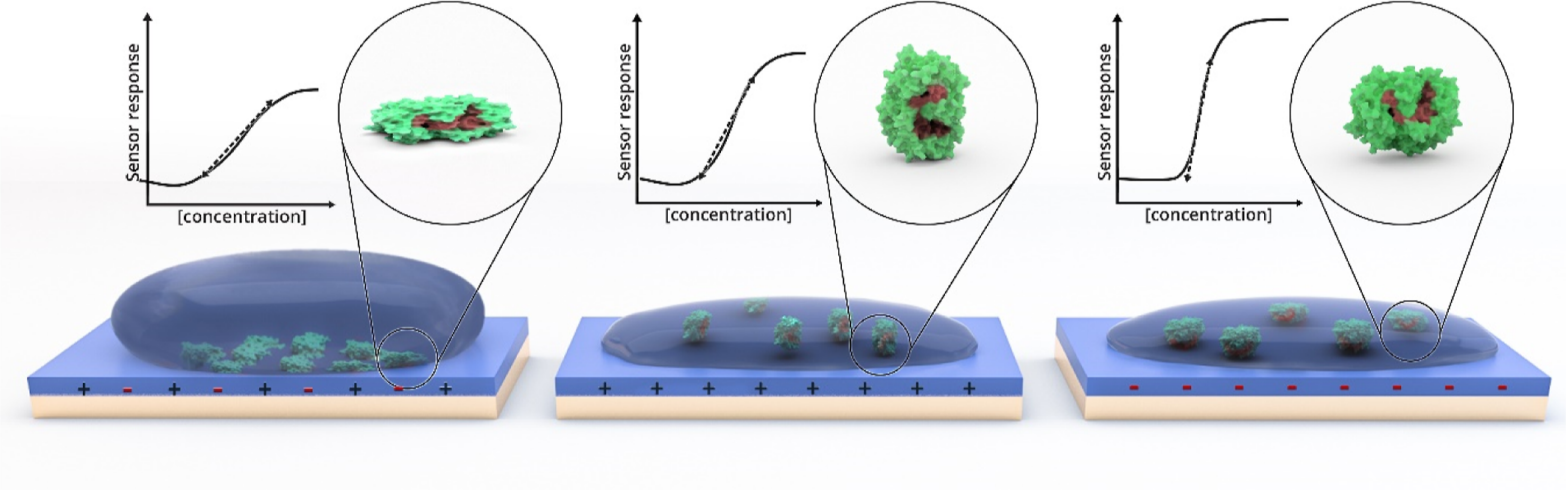
Influence of n-type polymer film surface properties on GOx adsorption. The surface hydrophilicity and charge govern enzyme adsorption behavior on the film surface. Hydrophobic surfaces tend to retain more enzymes. Surfaces that are too hydrophobic, however, lead to complete denaturation of the enzyme structure. GOx adopts a flattened conformation on hydrophilic/polar surfaces. The surface charge influences GOx orientation. A neutral surface (left) leads to a “front-lying” orientation, where the active site of the enzyme faces downward and is inaccessible to the glucose. A positively charged surface (middle) leads to the enzyme adsorbing in a “standing” orientation, where the active site is accessible. A negatively charged surface (right) leads to a “back-lying” orientation, with the active site facing up and in closer proximity to the sensor surface than in a standing fashion. The latter surface, which is hydrophilic, negatively charged, and homogeneous, is most desirable to build top-performer glucose sensors.

Our study points out the following conclusions: (i) GOx has different packing arrangements and conformations depending on the properties of each polymer surface. It adopts an expanded conformation on polar/hydrophilic surfaces and undergoes partial denaturation on hydrophobic surfaces; (ii) on hydrophobic surfaces, there is more enzyme, independent of the surface charge; (iii) the rigidity of the GOx layer decreases with the EG content of the surface, exhibiting a looser and flatter layer on more hydrophilic/polar surfaces; (iv) the orientation and conformation adopted upon adsorption, mainly dictated by the surface charge and hydrophobicity (see Supporting Information Discussion 1), influence the enzyme activity, indicating the need for a possible conformation change (flattening) to interact electrochemically with the underlying semiconducting film. We have summarized our findings in Figure 7.

## Conclusions

In this work, we investigated electronic film surface properties for the adsorption of an oxidase enzyme to achieve high-performance metabolite sensors. We correlated the sensor performance with the protein adsorption behavior by systematically probing the polymer surface properties and enzyme/polymer interactions. The nature of the conjugated polymer side chains governed the film surface properties and played a critical role in the enzyme orientation, conformation, and adsorption behavior. Our study revealed that GOx adopted different arrangements upon adsorption on each surface, influenced by the surface hydrophilicity and charge, while the morphology and roughness did not play a significant role. Hydrophobic surfaces retained more enzymes upon adsorption, where the protein presented a more rigid and densely packed arrangement with a smaller footprint than that on hydrophilic surfaces. However, increasing the hydrophobicity of surfaces led to the partial or total denaturation of the enzyme, thus decreasing the enzyme’s biological activity. We found that the enzymes specifically interacted with EG side chains, which were more favorable for the linear ones than the branched ones. The rigidity of the protein layer decreased with an increase in the EG content of the film surface, as we observed a looser and more flattened enzyme layer on more hydrophilic/polar surfaces. On the other hand, the performance of our sensors correlated with the orientation of GOx on the polymer surface (which was modulated by the surface charge), where a negatively charged surface led to the most favorable orientation for efficient catalysis.

Investigation of the surface chemical groups revealed an increased electron density around the C=C and C=O bonds upon enzyme adsorption, following the increase in the OECT-based sensor performance. These observations were supported by CD measurements, which showed that although GOx altered its conformation on all surfaces, it retained its native structure and, subsequently, activity on the polymers that made the best-performing sensors. Our studies suggest that a slightly negatively charged, smooth, and hydrophilic surface presents the best surface properties to maximize the sensor performance. This work establishes critical guidelines for designing conjugated polymers for mediator-free enzymatic metabolite sensors. Although these results could be applicable to other catalytic enzymes that share similar structural units with GOx, we note the robustness of GOx which may be playing a role in generating effective hybrids with n-type polymers. Although our core application concerns glucose sensors, our systematic analysis would benefit other enzyme-based bioelectronic applications, such as enzymatic biofuel cells and any organic electronic device that relies on protein/electronic material interactions.

## Supporting Information Available

Materials and methods; NMR spectrum of P-ZI; OECT output characteristics; O_2_ sensitivity of n-type films; water contact angle of n-type films; glucose oxidase surface charge distribution; AFM images of n-type films, QCM-D traces, models, and analysis of n-type films; list of amino acids in glucose oxidase; XPS signals of glucose oxidase adsorbed on n-type films and spectra deconvolutions; CD spectra of glucose oxidase in solution and when adsorbed on n-type films; discussions on the effect of surface charge and hydrophobicity on protein adsorption; and discussion on protein adsorption behavior on n-type films (PDF)
