# Supplementary material for: Interactions of Catalytic Enzymes with n-Type Polymers for High-Performance Metabolite Sensors
Source: ACS Appl Mater Interfaces. 2023 Feb 7;15(7):9726–39. doi: 10.1021/acsami.2c20502 (PMC9951220; doi:10.1021/acsami.2c20502)
Supplement: Supplementary file 1 — am2c20502_si_001.pdf [file am2c20502_si_001.pdf]

SUPPORTING INFORMATION

Interactions of catalytic enzymes with n-type polymers for high-performance metabolite sensors

David Ohayon,[1] Dominik Renn,[2] Shofarul Wustoni,[1] Keying Guo,[1] Victor Druet,[1] Adel Hama,[1] Xingxing Chen,[3] Iuliana Petruta Maria,[4] Saumya Singh,[5] Sophie Griggs,[4] Bob C. Schroeder,[5] Magnus Rueping,[2] Iain McCulloch,[3,4] Sahika Inal[1*]

[1] Organic Bioelectronics Laboratory, Biological and Environmental Science and Engineering Division, King Abdullah University of Science and Technology (KAUST), Thuwal 23955-6900, Saudi Arabia.

[2] Catalysis Center, King Abdullah University of Science and Technology (KAUST), Thuwal 23955-6900, Saudi Arabia.

[3] Physical Science and Engineering Division, KAUST, Thuwal 23955-6900, Saudi Arabia.

[4] Department of Chemistry, University of Oxford, Oxford OX1 3TA, U.K.

[5] Department of Chemistry, University of College London, 20 Gordon Street, London WC1H 0AJ, United Kingdom.

*Corresponding author: sahika.inal@kaust.edu.sa

Synthesis of the polymers

All reactions were performed in oven-dried glassware under a nitrogen atmosphere using standard Schlenk techniques unless otherwise stated. Monomer (3,3'-bis(2-(2-(2-methoxy ethoxy)ethoxy)ethoxy)-[2,2'-bithiophene]-5,5'-diyl)bis(trimethyl stannane) was synthesized as previously reported.[1] All commercially available materials were used as received. ^{1}H and ^{13}C spectra were recorded on Bruker AV-400 and Bruker AVIII HD 500 spectrometers at ambient temperature unless otherwise stated. Chemical shifts are reported in units of parts per million (ppm, δ) for solutions in either chloroform-*d*, trifluoroacetic acid-*d,* or hexafluoroisopropanol-d_2, and coupling constants (J) are given in Hz. The chemical shifts were referenced using residual chloroform (^{1}H NMR: 7.26 ppm, ^{13}C NMR: 77.36 ppm), trifluoroacetic acid (^{1}H NMR: 11.50 ppm, ^{13}C NMR: 164.2 and 116.6 ppm), or hexafluoroisopropanol-d_2 (^{1}H NMR: 4.86 ppm) peaks as an internal standard. Number average (M_n) and weight average (M_w) molecular weights in chloroform at 40 °C were determined using an Agilent Technologies 1260 infinity GPC at 40 °C, using two PLgel 10-micrometer Mixed-B columns in series (300 × 7.5 mm), and calibrated against narrow weight-average dispersity ($Đ$ < 1.10) polystyrene standards.

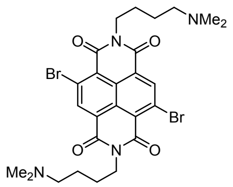

NDI-C4-DMA. *N,N*-Dimethyl-1,4-butanediamine (0.22 g, 1.88 mmol, 2.0 equiv) was added to a suspension of 2,6-dibromonaphthalene-1,4,5,8-tetracarboxydianhydride (0.40 g, 0.94 mmol, 1.0 equiv) in propionic acid (10 mL) and the resulting mixture was heated to 120 °C for 2 h. The reaction mixture was poured over ice and treated with a saturated aqueous $NaHCO_3$ solution until a pH of 8 was obtained. The resulting mixture was extracted with chloroform (3 × 200 mL), and

the combined organic portions were washed with water (3 × 200 mL) and dried over anhydrous $MgSO_4$. The solvent was removed *in vacuo,* and the solid residue was washed with MeOH and hot acetone to give the title product as an orange solid (0.14 g, 24%). ^{1}H NMR (400 MHz, trifluoroacetic acid-*d*) δ 9.77 (s, 2H), 5.03 (t, J = 7.3 Hz, 4H), 4.04 (t, J = 7.6 Hz, 4H), 3.73 (s, 12H), 2.73 – 2.59 (m, 8H). ^{13}C NMR (100 MHz, trifluoroacetic acid-*d*) δ 165.2, 142.4, 132.2, 130.2, 127.2, 126.6, 61.0, 45.7, 43.0, 26.2, 24.2.

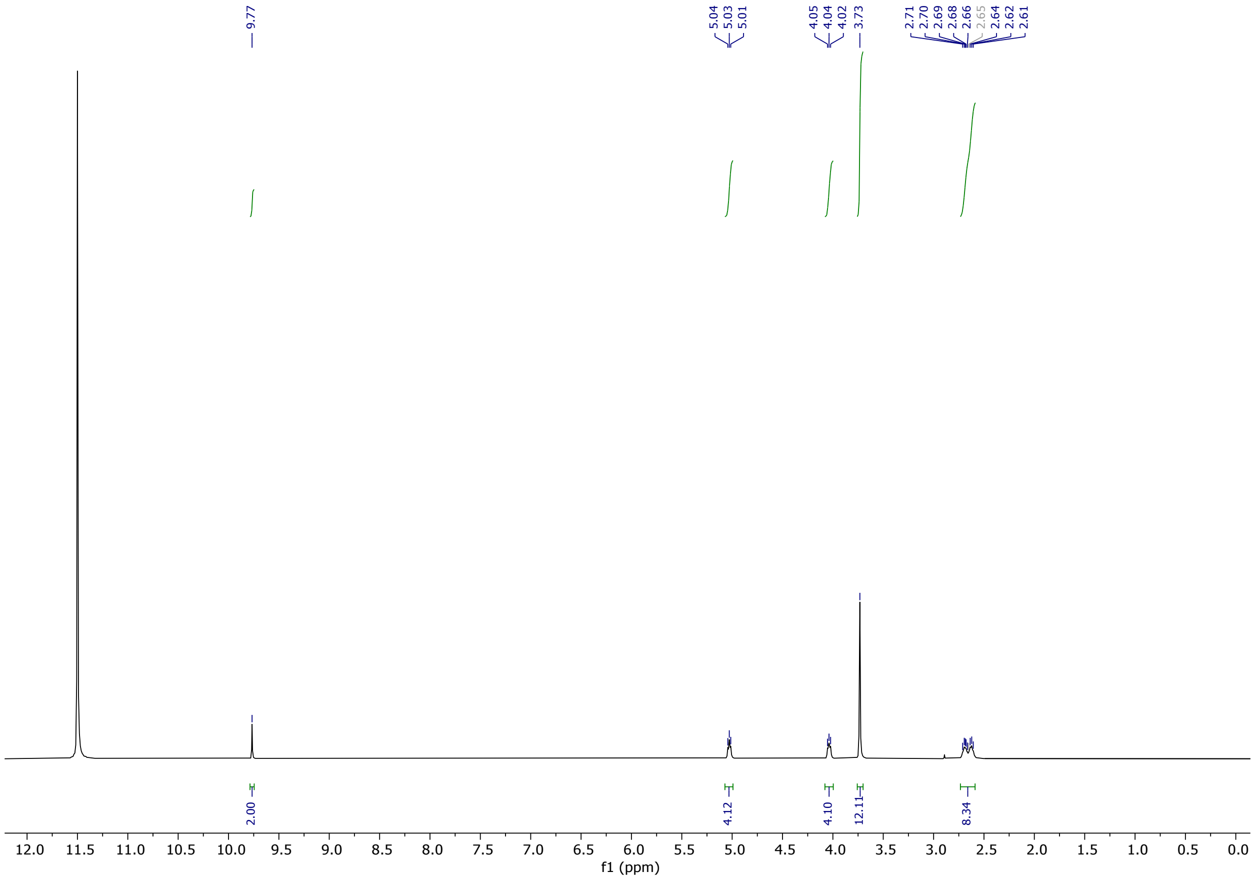

Figure S1. ^{1}H NMR (500 MHz) spectrum of **NDI-C4-DMA** in trifluoroacetic acid-*d*.

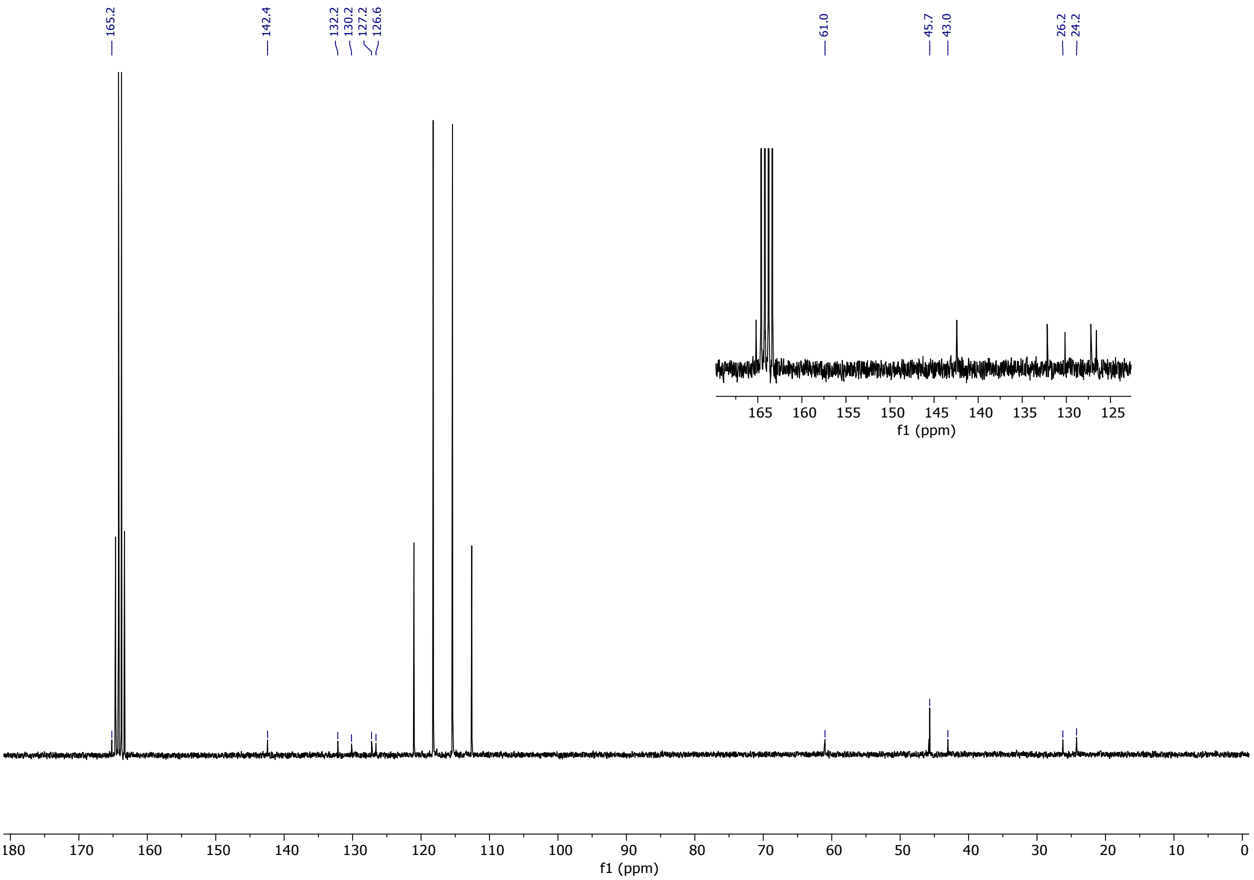

Figure S2. ^{13}C NMR (400 MHz) spectrum of **NDI-C4-DMA** in trifluoroacetic acid-*d*.

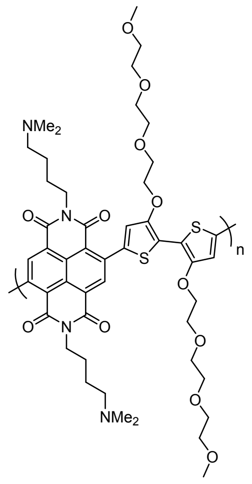

C4-DMA. A mixture of **NDI-C4-DMA** (35.13 mg, 56.45 μmol, 1 Equiv), (3,3'-bis(2-(2-(2-methoxyethoxy)ethoxy)ethoxy)-[2,2'-bithiophene]-5,5'-diyl)bis(trimethyl stannane) (46.08 mg, 56.45 μmol, 1.0 equiv), tris(dibenzylideneacetone)-dipalladium(0) (1.03 mg, 1.13 μmol, 0.02 equiv) and tri(*o*-tolyl)phosphine (1.37 mg, 4.52 μmol, 0.08 equiv) in anhydrous, degassed DMF (1.5 mL) was heated to 85 °C for 16 h. A solution (0.1 mL) of 2-(tributylstannyl)thiophene (0.1 mL) and $Pd_2(dba)_3$ (1.05 mg, 1.15 μmol) in anhydrous, degassed chlorobenzene (0.5 mL) was added and the resulting mixture was stirred for one h at 90 °C. A solution (0.1 mL) of 2-bromothiophene (0.1 mL) in anhydrous, degassed chlorobenzene (0.5 mL) was subsequently added, and stirring at 90 °C was maintained for a further one h. The reaction mixture was cooled to room temperature and precipitated in ethyl acetate, followed by adding hexane. The solid was collected in a glass thimble and subjected to Soxhlet extractions with hexane, ethyl acetate, MeOH, acetone, and chloroform. After the final extraction with chloroform, the polymer solution was concentrated *in vacuo* and precipitated in ethyl acetate, followed by the addition of hexane. The polymer was collected by filtration and dried under reduced pressure to afford the product as a green solid with a yield of 51% (27.3 mg, 28.7 μmol). ^1H NMR (400 MHz, chloroform-*d*) δ 8.84 (br s, 2H), 4.54 – 4.29 (m, 8H), 4.00 (m, 4H), 3.83 – 3.79 (m, 4H), 3.70 – 3.67 (m, 4H), 3.62 (m,

4H), 3.51 (d, J = 5.4 Hz, 4H), 3.36 – 3.31 (m, 6H), 2.38 (m, 4H), 2.28 (br s, 12H), 1.85 (m, 4H), 1.64 (s, 4H). GPC (chloroform, 40 °C).

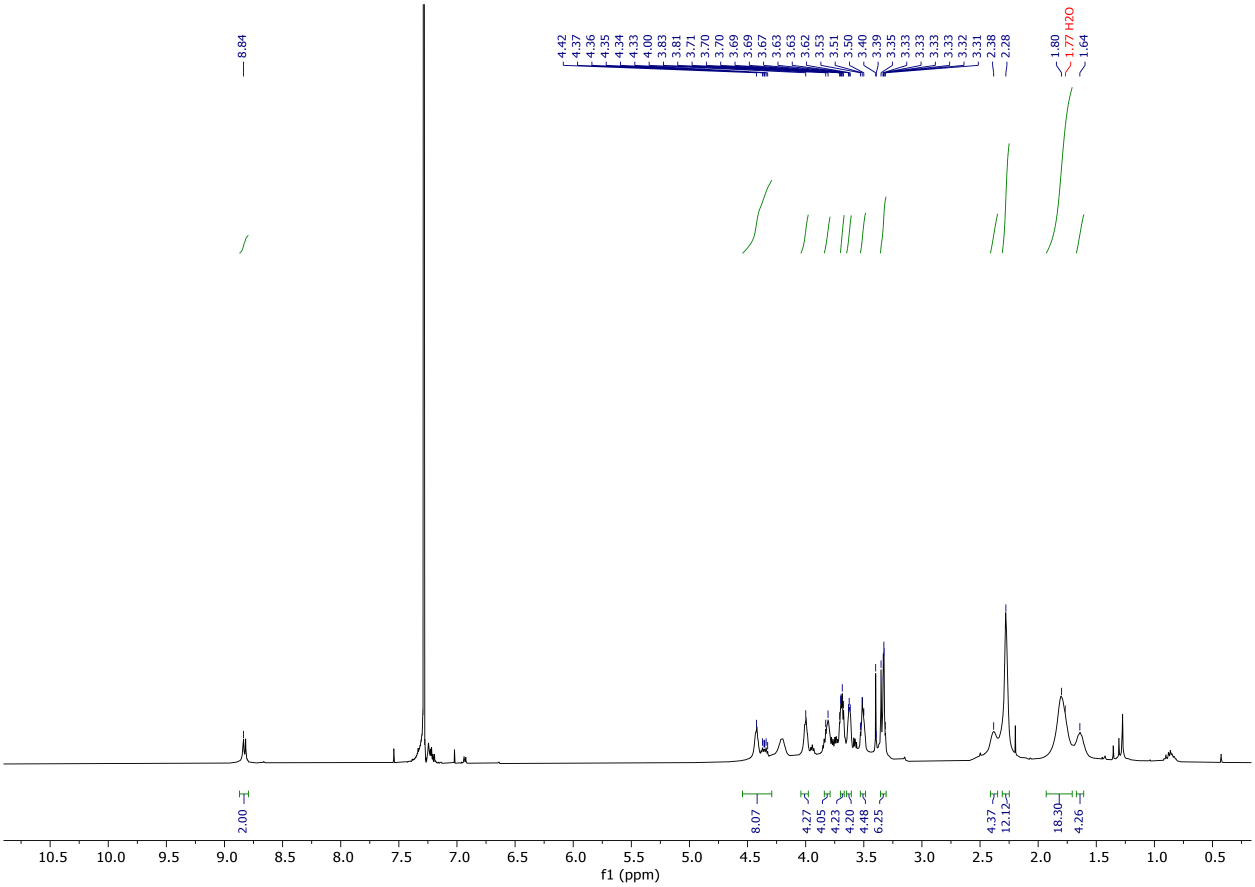

Figure S3. ^{1}H NMR (400 MHz) spectrum of **C4-DMA** in chloroform-*d*.

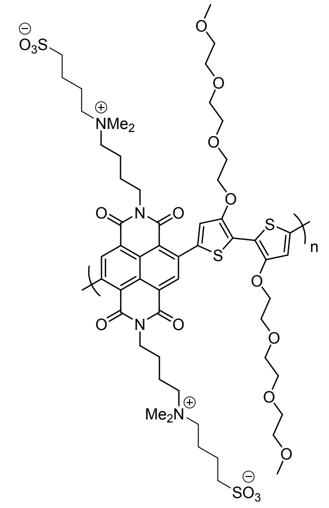

C4-ZI. 1,4-Butane sultone (0.6 mL) was added to a suspension of **C4-DMA** (15.0 mg, 15.8 μmol) in anhydrous DMF (4 mL), and the resulting mixture was heated to 120 °C for 16 h. The reaction mixture was allowed to reach room temperature, and the solvent was removed under a vacuum. The green residue was suspended in acetone, filtered, and thoroughly washed with hot acetone and chloroform to give the title product as a green solid with a yield of 64% (12.4 mg, 10.1 μmol). ^{1}H NMR (400 MHz, hexafluoroisopropanol-d_2) δ 8.95 (br s, 2H), 7.26 (br s, 2H), 4.50 – 4.40 (m, 8H, overlapped with residual solvent peak), 4.02 – 3.59 (m, 20H), 3.52 – 3.47 (m, 6H), 3.32 – 3.24 (m, 4H), 3.15 – 2.97 (m, 20H), 2.06 – 1.78 (s, 16H).

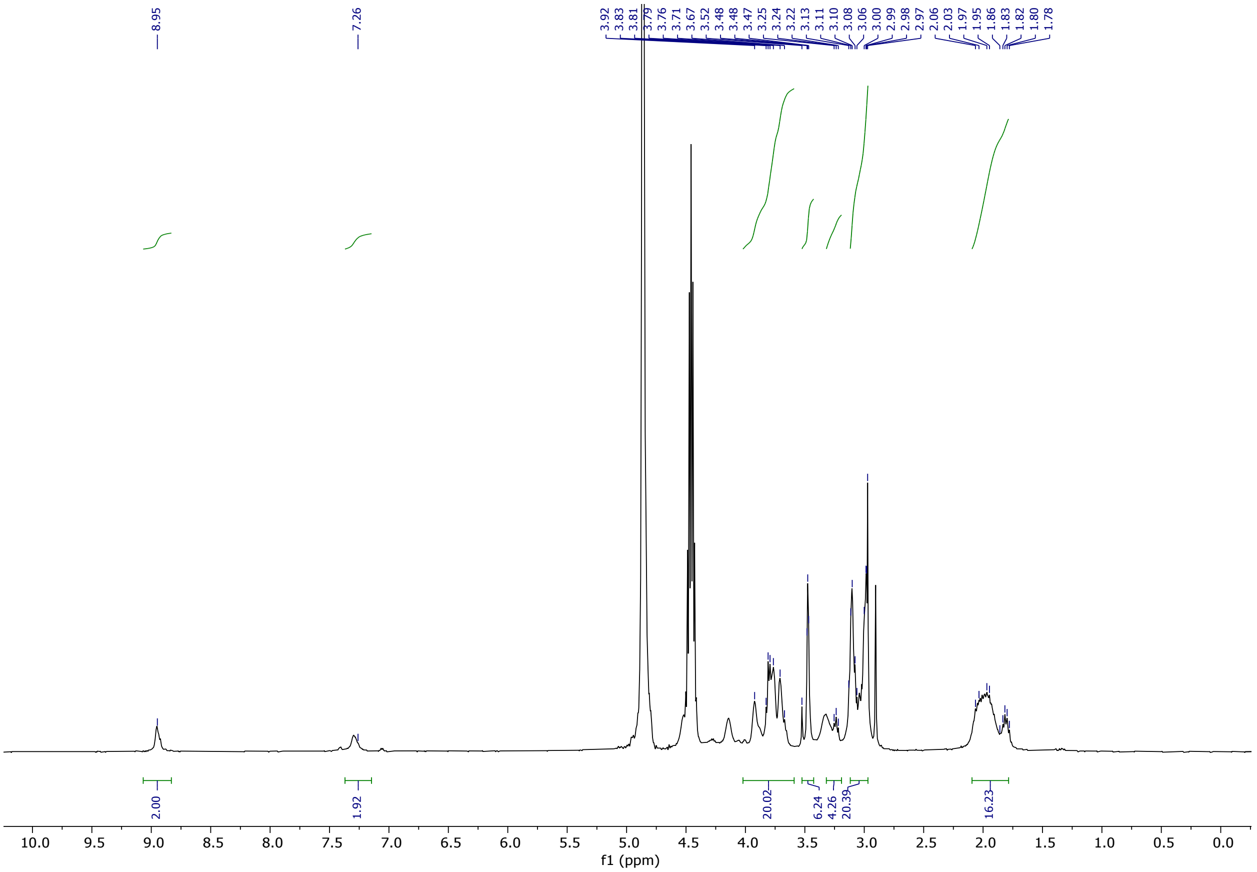

Figure S4. ^{1}H NMR (400 MHz) spectrum of **C4-ZI** in hexafluoroisopropanol-d_2.

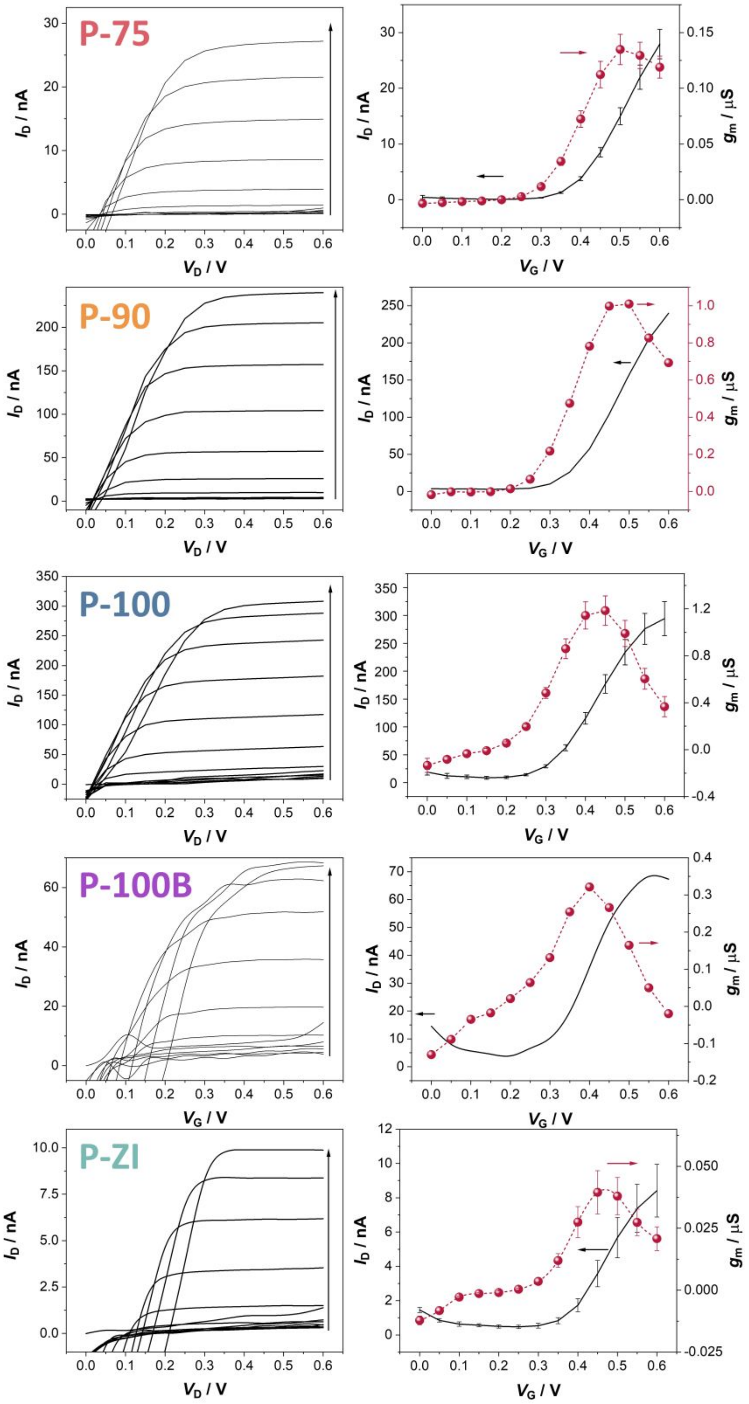

Figure S5. OECT output and transfer curves using an Ag/AgCl gate electrode. The arrows in the output curves indicate the increase in the gate voltage from 0 to 0.6 V. All channels have the same dimensions, and the film thickness ranges from 60 to 80 nm.

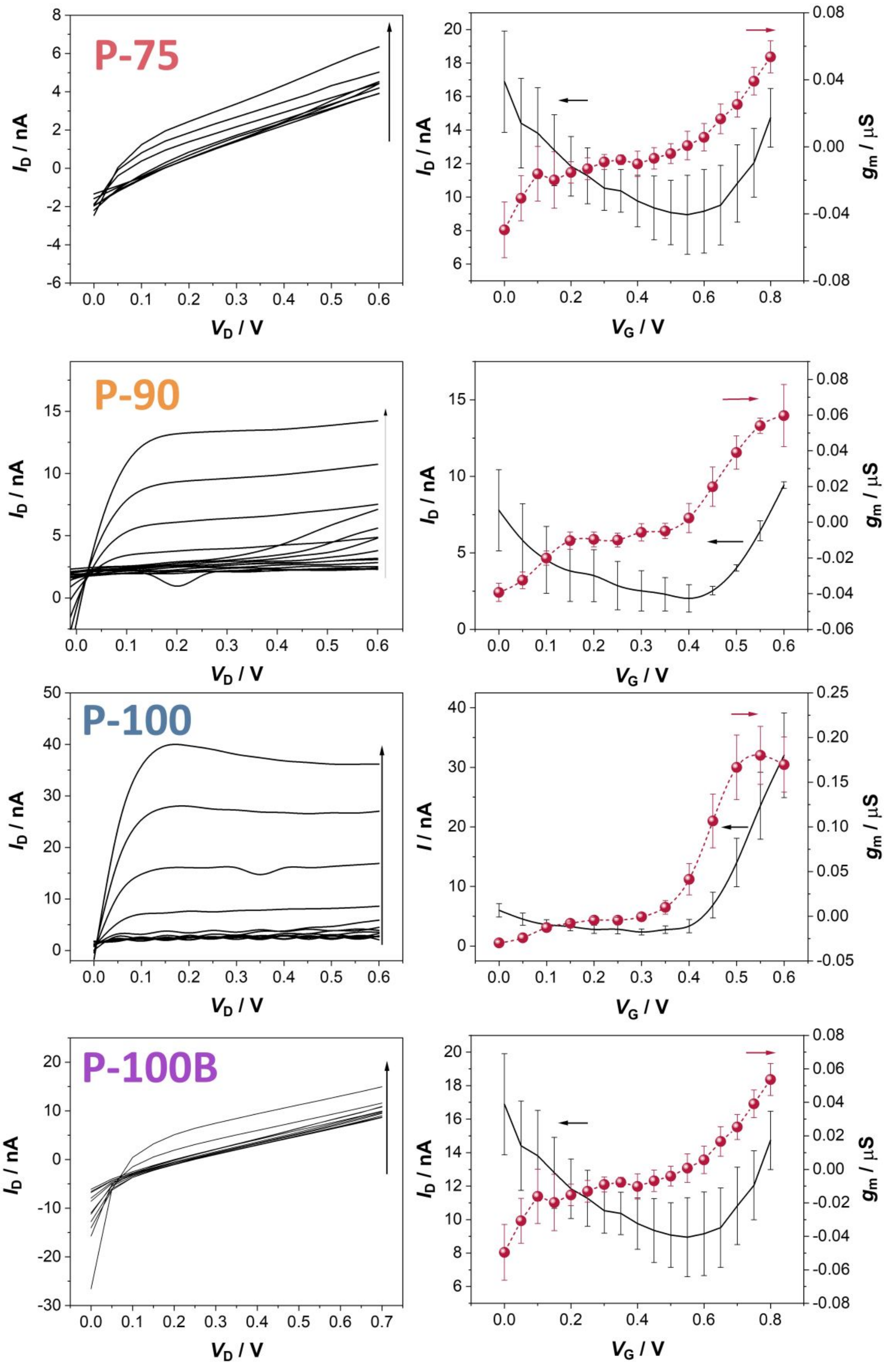

Figure S6. OECT output and transfer curves using an n-type film-coated Au gate electrode. The arrows in the output curves indicate the increase in the gate voltage. P-ZI could not be operated using this configuration.

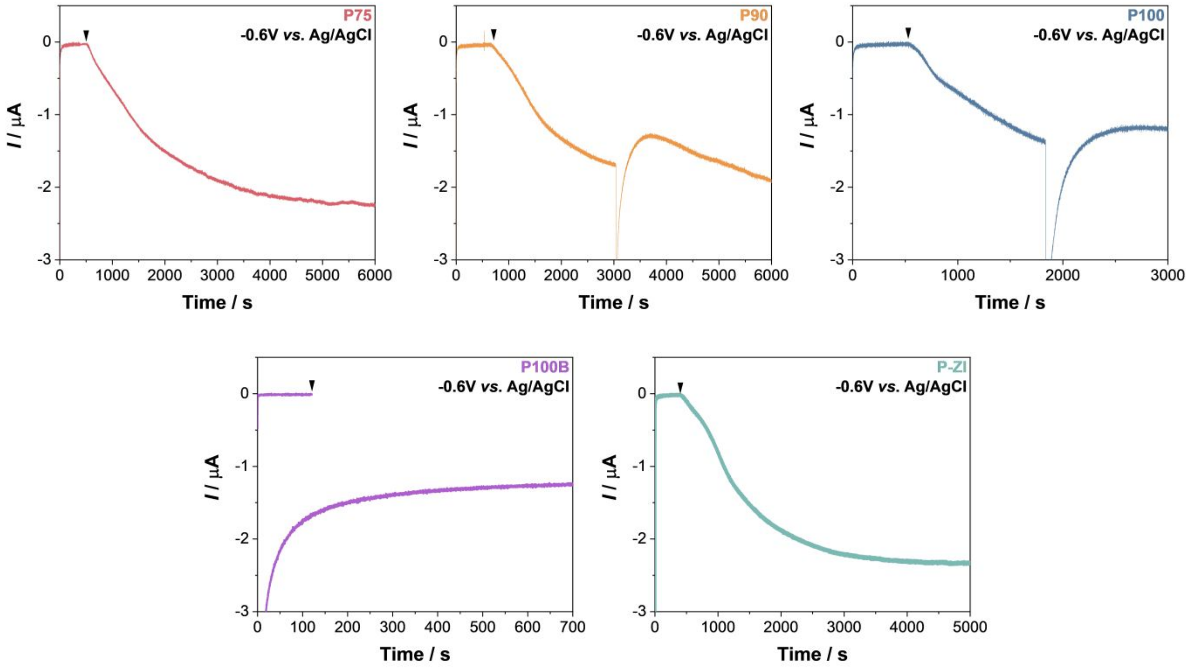

Figure S7. O_2 sensitivity of n-type films. The current response of n-type polymer-coated Au electrodes to O_2 was evaluated using a three-electrode setup. The working electrode was the GOx adsorbed n-type film, the reference electrode was Ag/AgCl, and the counter electrode was a Pt coil. The electrode was first immersed in a de-gassed electrolyte in a glovebox under an N_2-saturated atmosphere. Once a stabilized current was achieved, the glovebox door was opened (represented by the arrow) to allow the ambient O_2 to enter and dissolve in the electrolyte.

For P-90 and P-100, the dip in current corresponds to the pipetting of fresh electrolyte into the system. For P-100B, two different films were used (one measured in N_2, the other in ambient) as the film was delaminating when biased for an extended period.

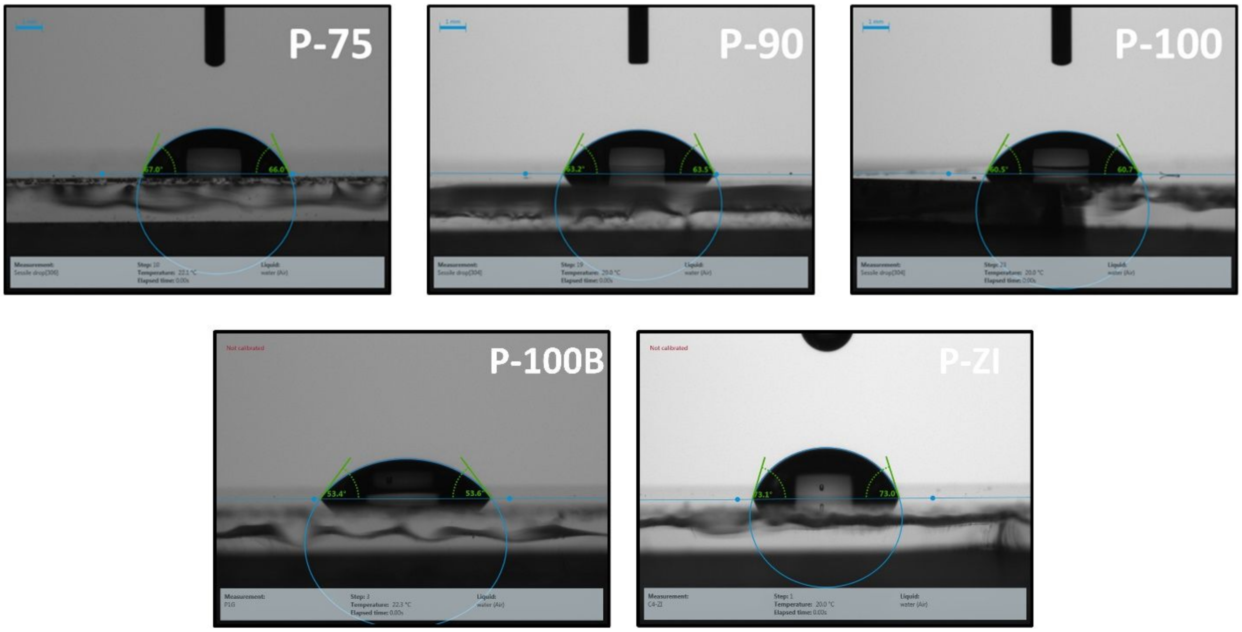

Figure S8. The water contact angle investigations on polymer films.

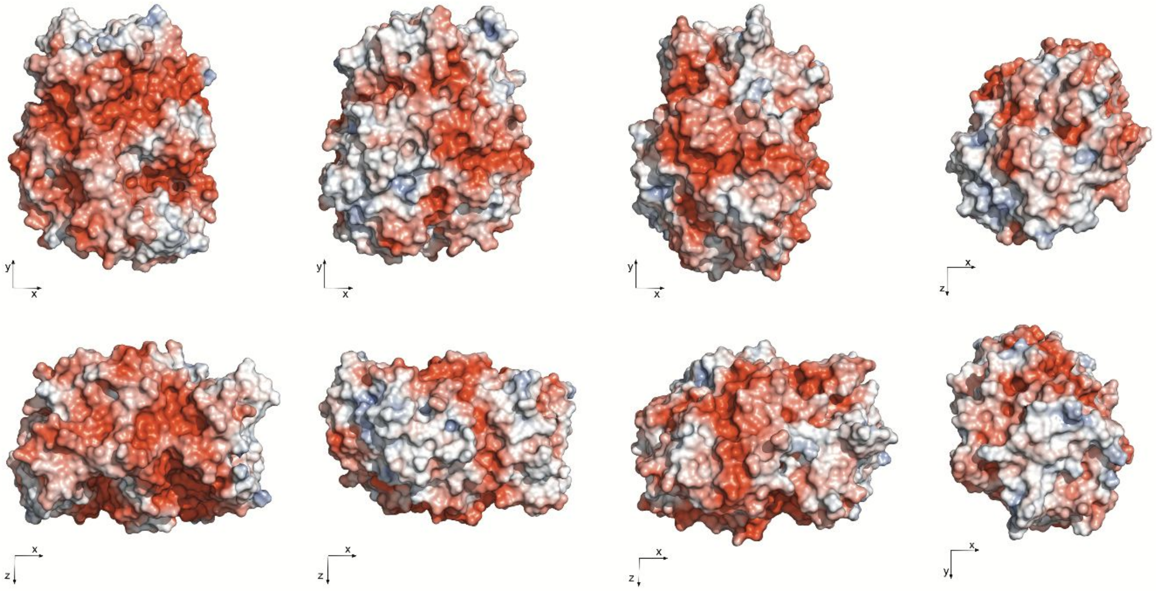

Figure S9. Surface charge representation of Glucose oxidase from *Aspergillus niger* (PDB: 3QVP) in its native state. Surface colors indicate positive and negative electrostatic potentials contoured from 50 kT/e (blue) to -50 kT/e (red) and visualized by PyMOL Molecular Graphics System, Version 2.4.2, Schrödinger, LLC.

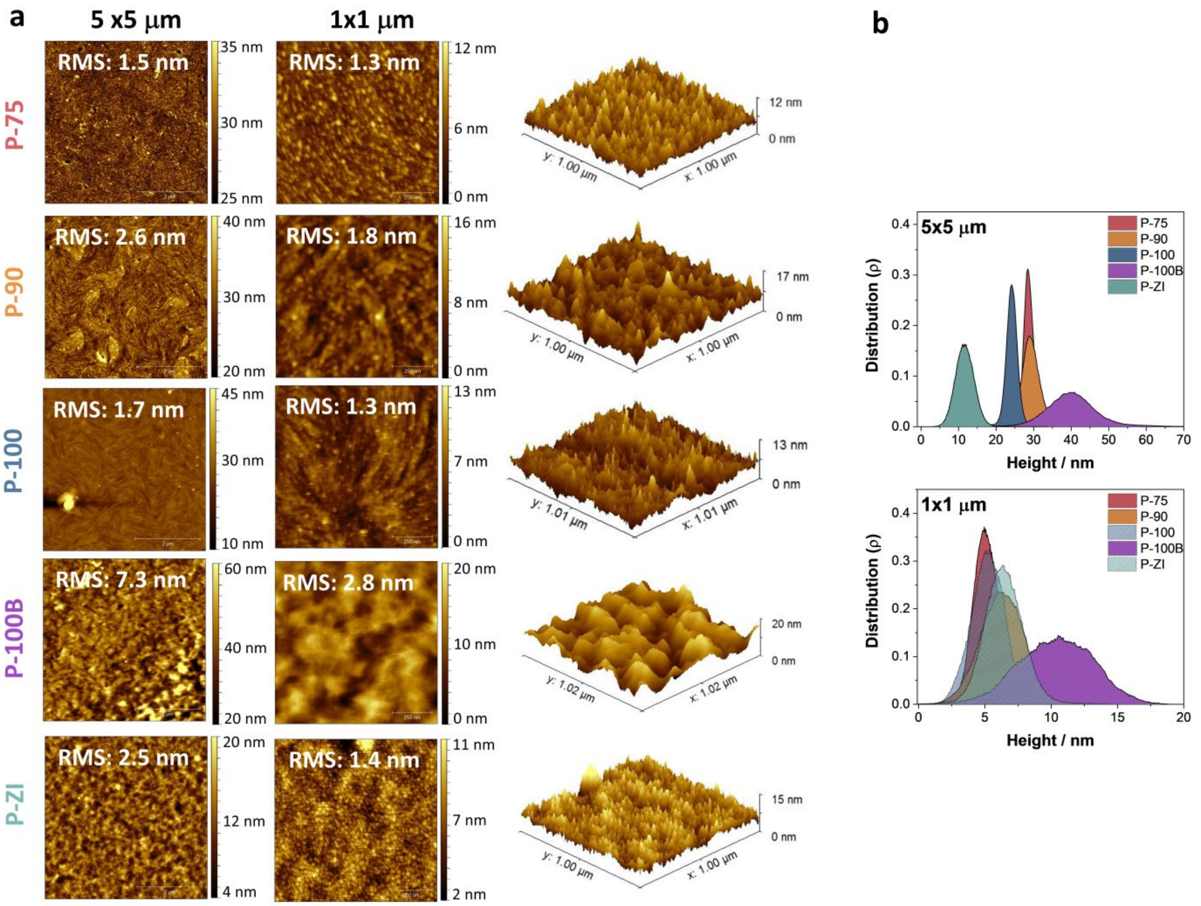

Figure S11. AFM topography images of n-type films immersed in PBS and their height distribution fraction. (**a**) Surface morphology images at 5 μm x 5 μm and 1 μm x 1 μm scale, with 3D representation shown at the right-hand side; (**b**) Corresponding relative height distribution.

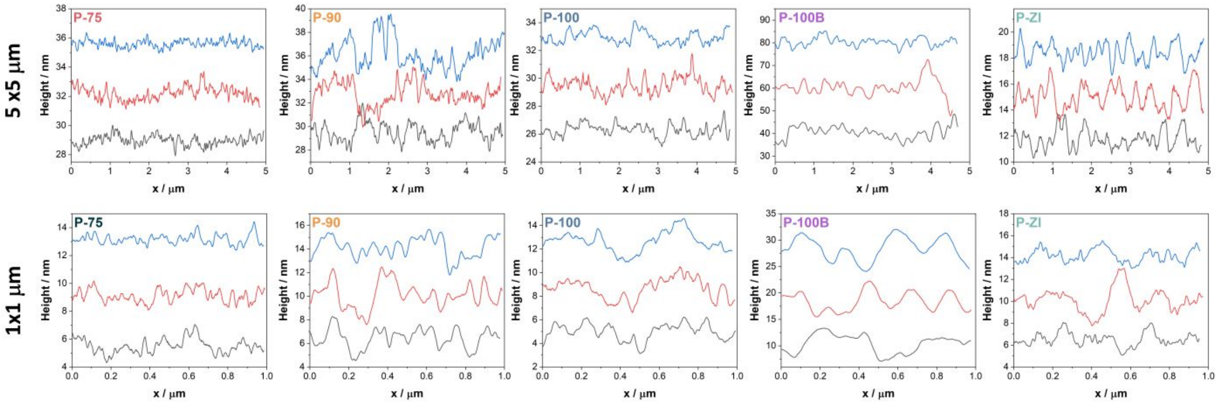

Figure S12. AFM lateral surface profile of n-type films immersed in PBS. The lines in the surface profiles were taken at three different locations and are represented with a Y-offset for clarity.

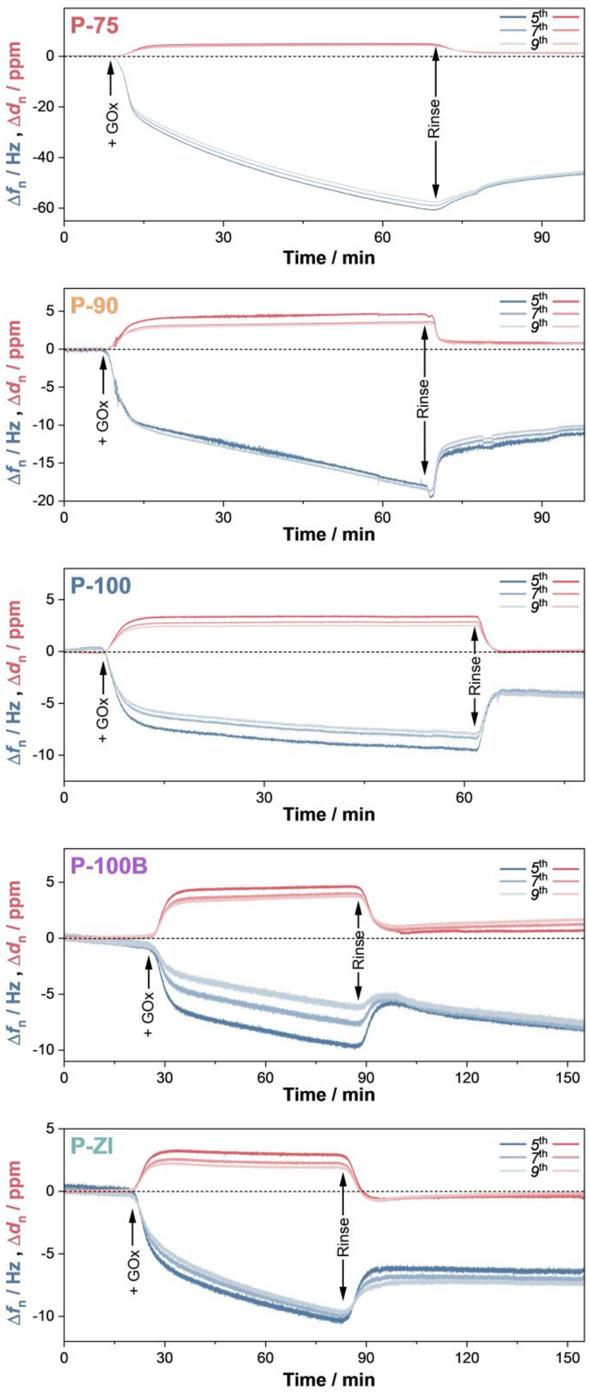

Figure S13. QCM-D measurements of GOx adsorption on the polymer films. The change in frequency (Δf_n) and the right one of the dissipation (Δd_n) for harmonics 5th, 7th, and 9th (n represents the harmonic number) are shown. After the films were fully swollen in PBS (no change in QCM-D signals), GOx was introduced to the chamber ("+ Gox"). After an hour of adsorption, the films were rinsed with PBS to remove any unbound species ("Rinse").

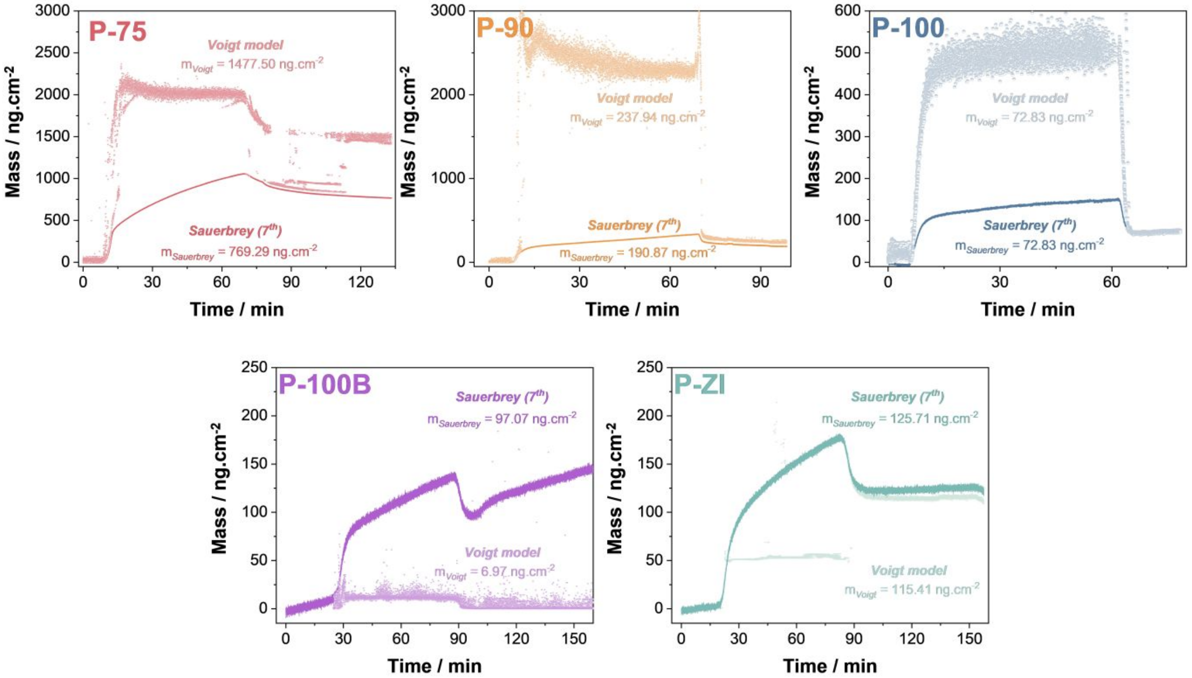

Figure S14. Application of the Voigt model. Voigt model fit (symbols) and the on the 5th, 7th, and 9th harmonic for the frequency and dissipation traces (lines). The adsorbed mass values are calculated from the Sauerbrey equation and the Voigt model.

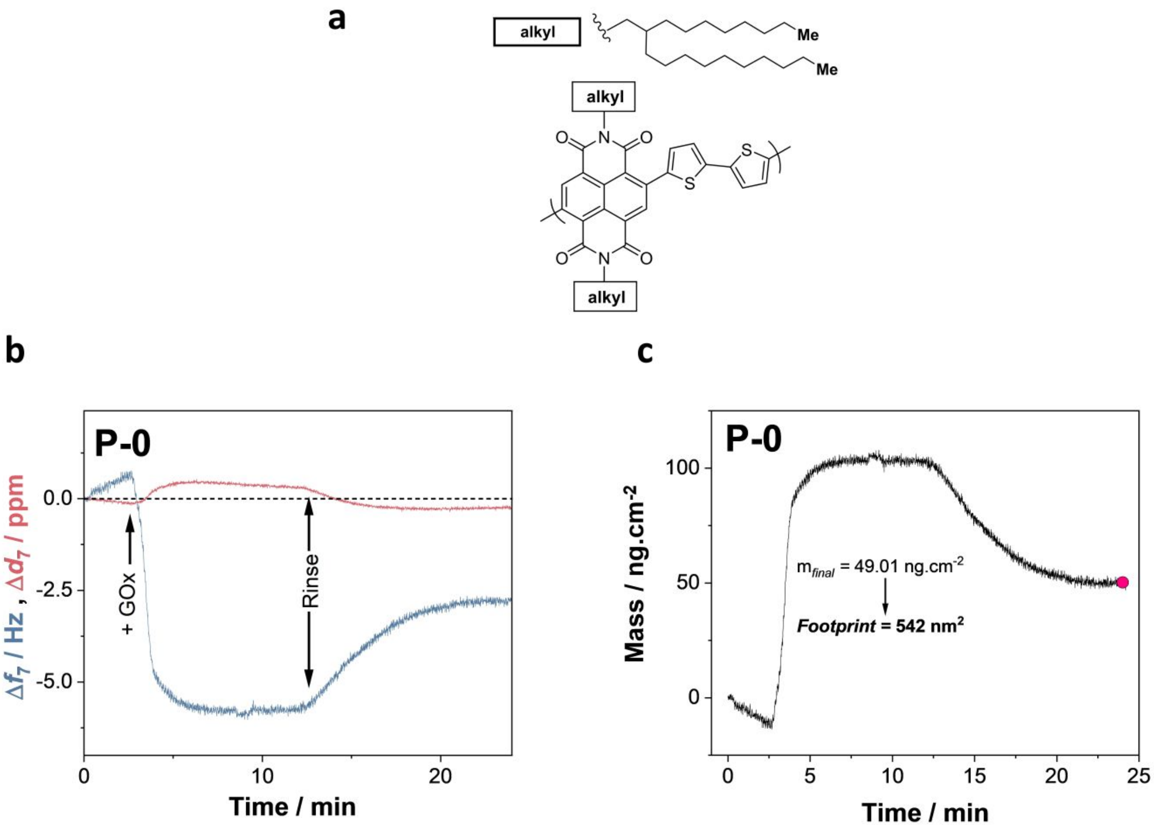

Figure S15. QCM-D measurements and analysis of GOx adsorption on P-0. (**a**) Chemical structure of P-0; (**b**) Raw data: the left axis reports the change in frequency (Δf_7) and the right one of the dissipation (Δd_7) for the 7th harmonic. P-0 film stabilized in PBS, after which we introduced GOx. After an hour of incubation with GOx, the film was rinsed with PBS; (**c**) The corresponding mass was taken up upon GOx adsorption. The adsorbed mass per area was extracted from the data collected at the end of the rinsing process (red dot).

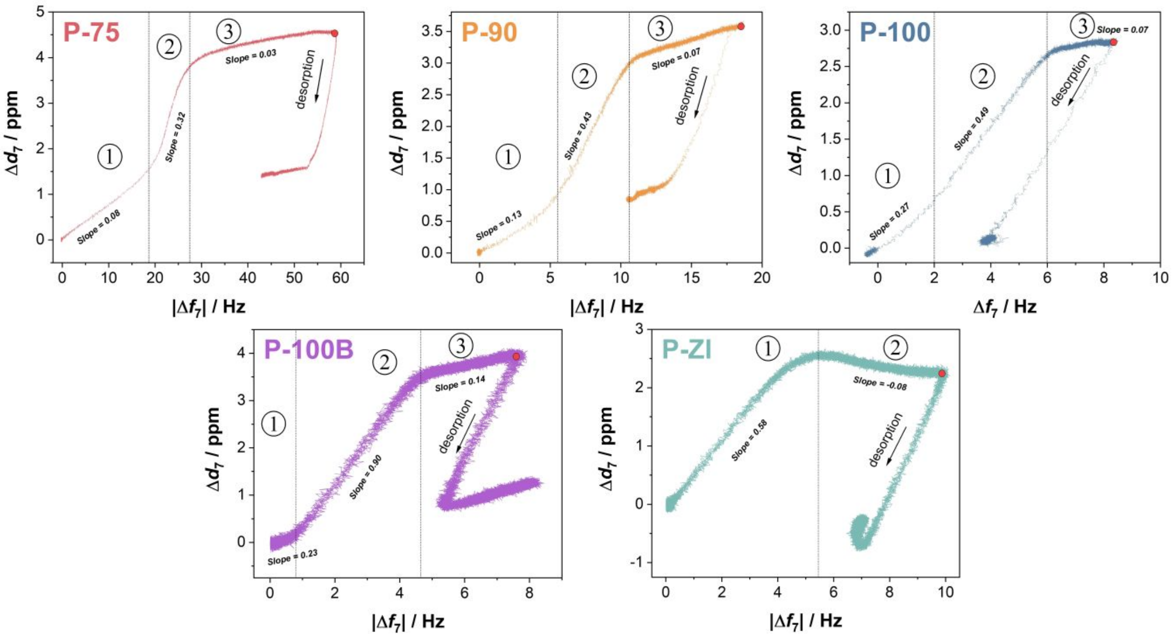

Figure S16. Probing the viscoelastic properties of the GOx layer on the polymer films. Δd vs. Δf plots (7th harmonic). The numbers define the linear regions in the plots with different slopes. The red dot demarks the transition from the adsorption process to the rinsing process.

Table S1. Δ*d* vs. Δ*f* slopes of GOx adsorption on polymers.

Slope *(Δd/Δf)* *10E-6	Phase ①	Phase ②	Phase ③
**P-75**	0.08	0.32	0.03
**P-90**	0.13	0.43	0.07
**P-100**	0.27	0.49	0.07
**P-100B**	0.23	0.90	0.14
**P-ZI**	0.58	-0.08	-

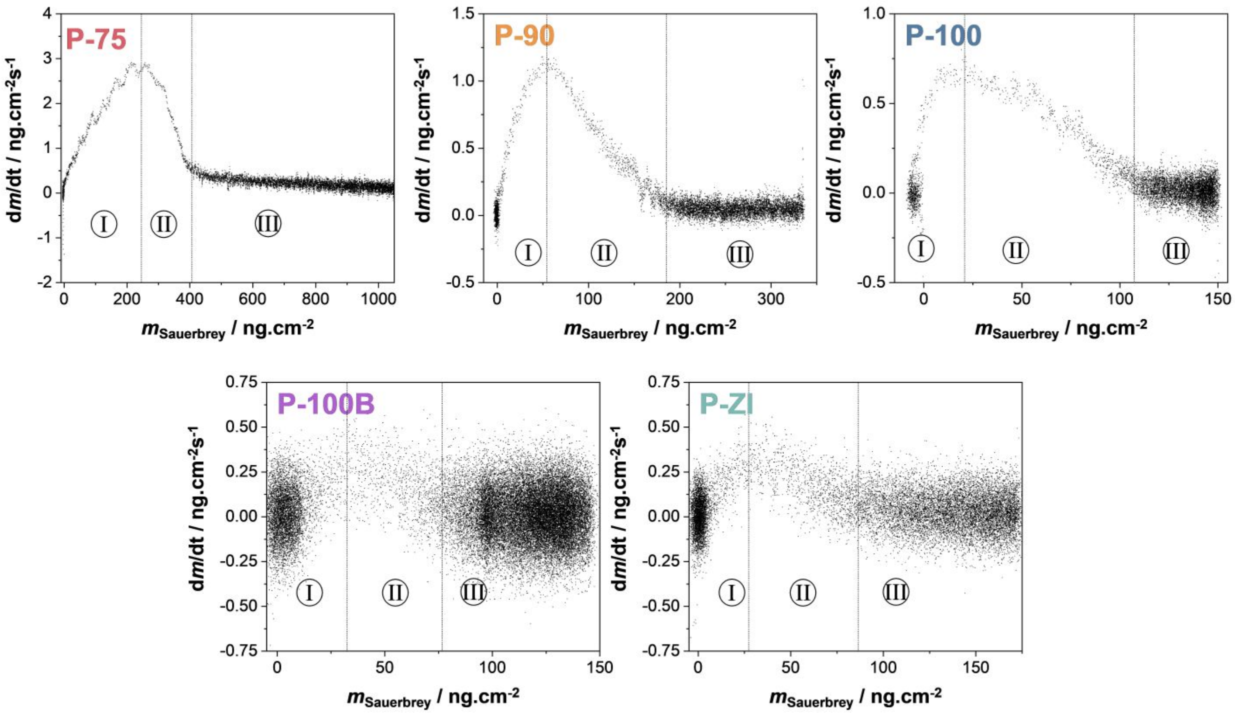

Figure S17. GOx adsorption kinetics. d*m*/d*t* vs. mass uptake plots for GOx adsorption on different polymer films. The calculations were made using the Sauerbrey model and the 7th harmonic.

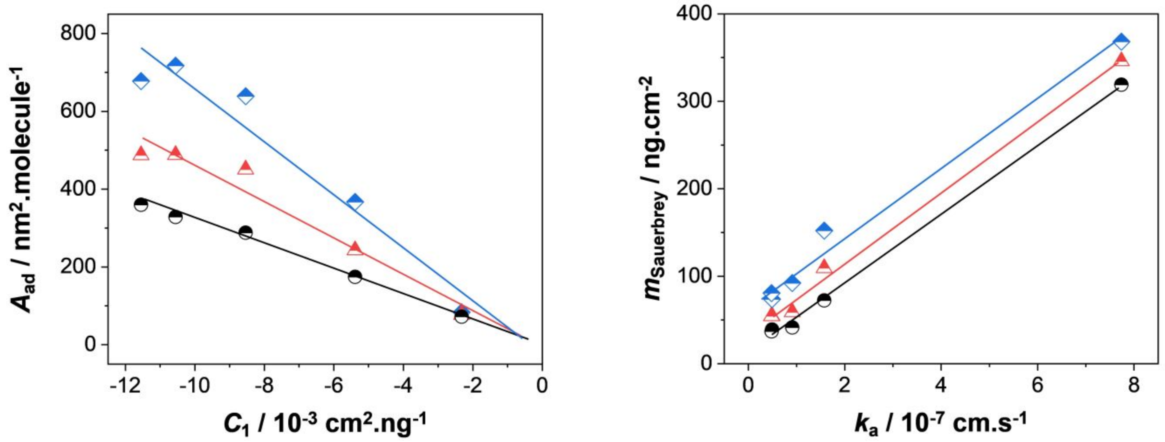

Figure S18. Relationship between Mass uptake, C_1, and k_a. A_{ad} represents the surface area the enzyme uses at a given time during its adsorption on each polymer. We sampled three arbitrarily chosen time points during the adsorption process to show the model's validity. Here, k_a is the rate constant of protein accumulation at low coverage, and C_1 reflects how the adsorbed protein molecules slow the subsequent adsorption of others.

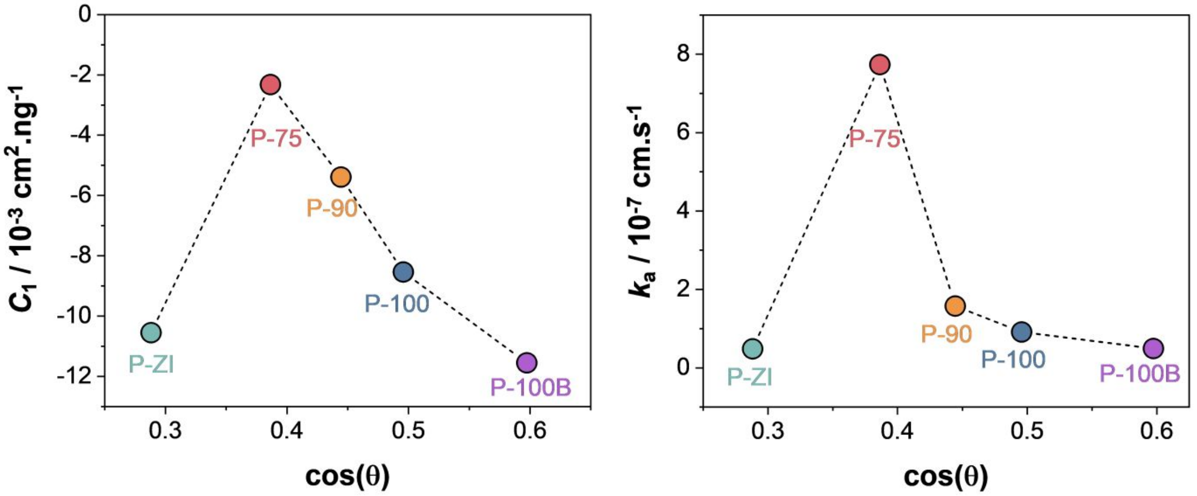

Figure S19. The relationship of C_1 and k_a with surface wettability.

Table S2. The deconvolution of the N 1s XPS spectra is shown in **Figure 6**.

**Bonds of interest**		**P-75**	**P-90**	**P-100**	**P-100B**	**P-ZI**
**$\underline{N}H_2$**	**B.E. (eV)**	399.900	399.700	399.761	399.867	399.900
	**Area**	6711.50	6703.5	9057.281	2660.478	3964.227
	**FWHM (eV)**	1.300				
**$\underline{N}$-C=O**	**B.E. (eV)**	400.170	400.000	400.455	400.099	400.183
	**Area**	807.28	1395.2	1509.172	739.908	927.093
	**FWHM (eV)**	0.995				
**$\underline{N}H_3^+$**	**B.E. (eV)**	401.000	401.000	401.164	401.498	401.000
	**Area**	475.46	232.649	1734.404	174.691	506.171
	**FWHM (eV)**	1.69				
**$\underline{N}$-Me_2^+**	**B.E. (eV)**	**-**	**-**	**-**	**-**	402.329
	**Area**	**-**	**-**	**-**	**-**	599.025
	**FWHM (eV)**	1.061				

B.E. = Binding energy
FWHM = Full width at half maximum

Table S3. Amino acid composition of glucose oxidase from *Aspergillus niger.* The amino acid sequence of glucose oxidase from *Aspergillus niger* (UniProtKB: P13006)[2] was analyzed by the Expasy server.[3-4]

**Amino Acid**	**Properties**	**% Composition**
Alanine (ALA)	Non-polar, aliphatic residues	10.1
Arginine (ARG)	Positively charged, polar, hydrophilic	3.8
Asparagine (ASN)	Polar, non-charged	5.5
Aspartate (ASP)	Negatively charged, polar, hydrophilic	6.0
Cysteine (CYS)	Polar, non-charged	0.5
Glutamine (GLN)	Polar, non-charged	3.8
Glutamate (GLU)	Negatively charged, polar, hydrophilic	5.0
Glycine (GLY)	Non-polar, aliphatic residues	9.4
Histidine (HIS)	Positively charged, polar, hydrophilic	3.3
Isoleucine (ILE)	Non-polar, aliphatic residues	4.5
Leucine (LEU)	Non-polar, aliphatic residues	8.9
Lysine (LYS)	Positively charged, polar, hydrophilic	2.5
Methionine (MET)	Polar, non-charged	2.0
Phenylalanine (PHE)	Aromatic	3.0
Proline (PRO)	Non-polar, aliphatic residues	4.3
Serine (SER)	Polar, non-charged	6.6
Threonine (THR)	Polar, non-charged	7.1
Tryptophan (TRP)	Aromatic	1.7
Tyrosine (TYR)	Aromatic	4.6
Valine (VAL)	Non-polar, aliphatic residues	7.6

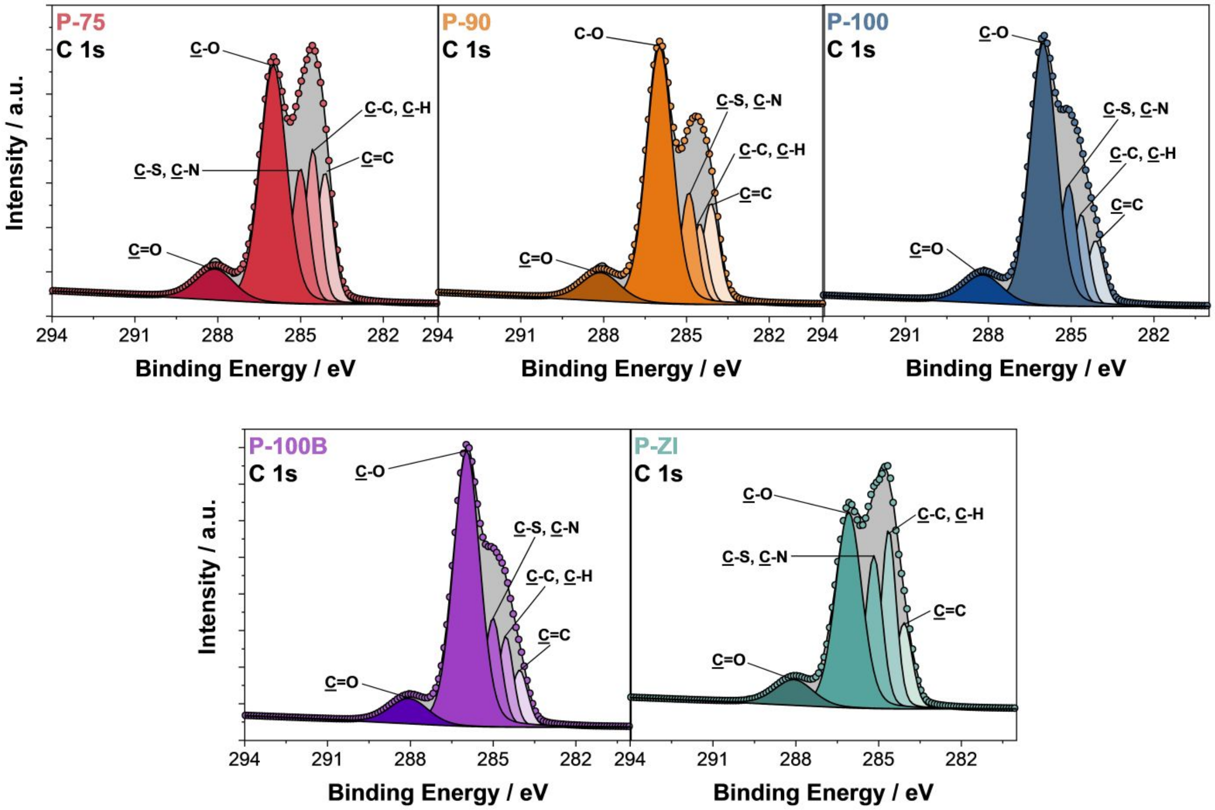

Figure S20. High-resolution of C 1s XPS spectra of polymer films.

Table S4. Deconvolution of the XPS spectra is shown in **Figure S20**.

Bonds of interest		P-75	P-90	P-100	P-100B	P-ZI
C=C	B.E. (eV)	284.117	284.115	284.118	284.044	284.081
	Area	4874.781	4780.36	2961.065	2560.433	3181.977
	FWHM (eV)	0.704				
C-C, C-H	B.E (eV)	284.555	284.52	284.634	284.543	284.636
	Area	5241.721	3329.191	3789.18	3736.844	6061.687
	FWHM (eV)	0.644				
C-S, C-N	B.E. (eV)	285.007	284.910	285.064	285.007	285.173
	Area	5158.502	5184.852	5652.145	5034.809	5288.42
	FWHM (eV)	0.726				
C-O	B.E. (eV)	285.986	285.871	286.012	285.960	286.083
	Area	14165.41	18538.74	18994.78	19919.99	11636.03
	FWHM (eV)	1.132				
C=O	B.E. (eV)	288.102	288.108	288.193	288.053	288.067
	Area	2661.328	2947.709	2938.752	2636.753	2210.407
	FWHM (eV)	1.638				

B.E. = Binding energy
FWHM = Full width at half maximum

Table S5. The relative contribution of C-C and C-O bonds in polymer films.

Polymers	C-C (%)	C-O (%)
**P-75**	16	44
**P-90**	10	53
**P-100**	11	55
**P-100B**	11	59
**P-ZI**	21	41

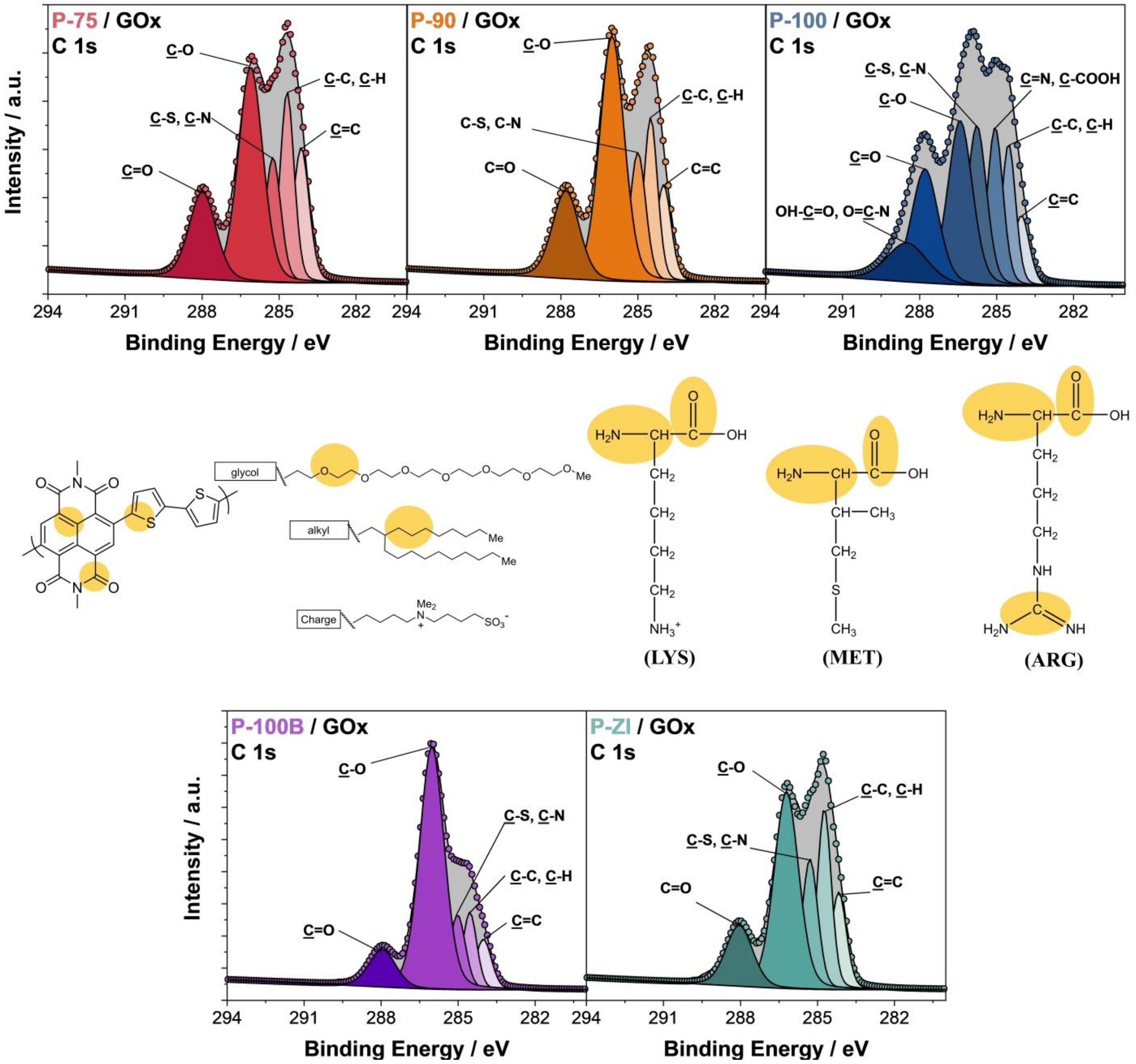

Figure S21. High-resolution of C 1s XPS spectra of the polymer films after enzyme adsorption. Yellow circles highlight the chemical bonds containing C atoms including the NDI backbone and side chains, and those in the primary amino acids present in GOx. The amino acids displayed in the figure correspond to only a selected few of the GOx sequence: lysine (LYS), methionine (MET), and arginine (ARG).

Table S6. Deconvolution of the C 1s XPS spectra is shown in **Figure S21**.

Bonds of interest		P-75	P-90	P-100	P-100B	P-ZI
**C=C**	**B.E. (eV)**	284.141	284.000	284.037	284.016	284.173
	**Area**	4844.427	3297.236	2093.77	2717.566	3374.901
	**FWHM (eV)**	0.741				
**C-C, C-H**	**E.B. (eV)**	284.687	284.502	284.531	284.545	284.734
	**Area**	5955.709	5087.776	3669.998	3626.688	5724.258
	**FWHM (eV)**	0.644				
**C-S, C-N**	**B.E. (eV)**	285.241	285	285.058	285.024	285.294
	**Area**	4391.257	4573.352	4607.676	3946.032	4644.64
	**FWHM (eV)**	0.726				
**C-COOH**	**B.E. (eV)**	**-**	**-**	285.768	**-**	**-**
	**Area**	**-**	**-**	5695.903	**-**	**-**
	**FWHM (eV)**	0.890				
**C-O**	**B.E. (eV)**	286.108	286.006	286.421	286.023	286.208
	**Area**	11736.24	13402.43	7421.731	19891.95	10917.3
	**FWHM (eV)**	1.130				
**C=O**	**B.E. (eV)**	287.984	287.804	287.787	287.951	288.046
	**Area**	5194.778	5242.722	5608.985	3341.316	3689.402
	**FWHM (eV)**	1.232				
**-COOH**	**B.E. (eV)**	**-**	**-**	288.498	**-**	**-**
	**Area**	**-**	**-**	2835.863	**-**	**-**
	**FWHM (eV)**	1.820				

B.E. = Binding energy
FWHM = Full width at half maximum

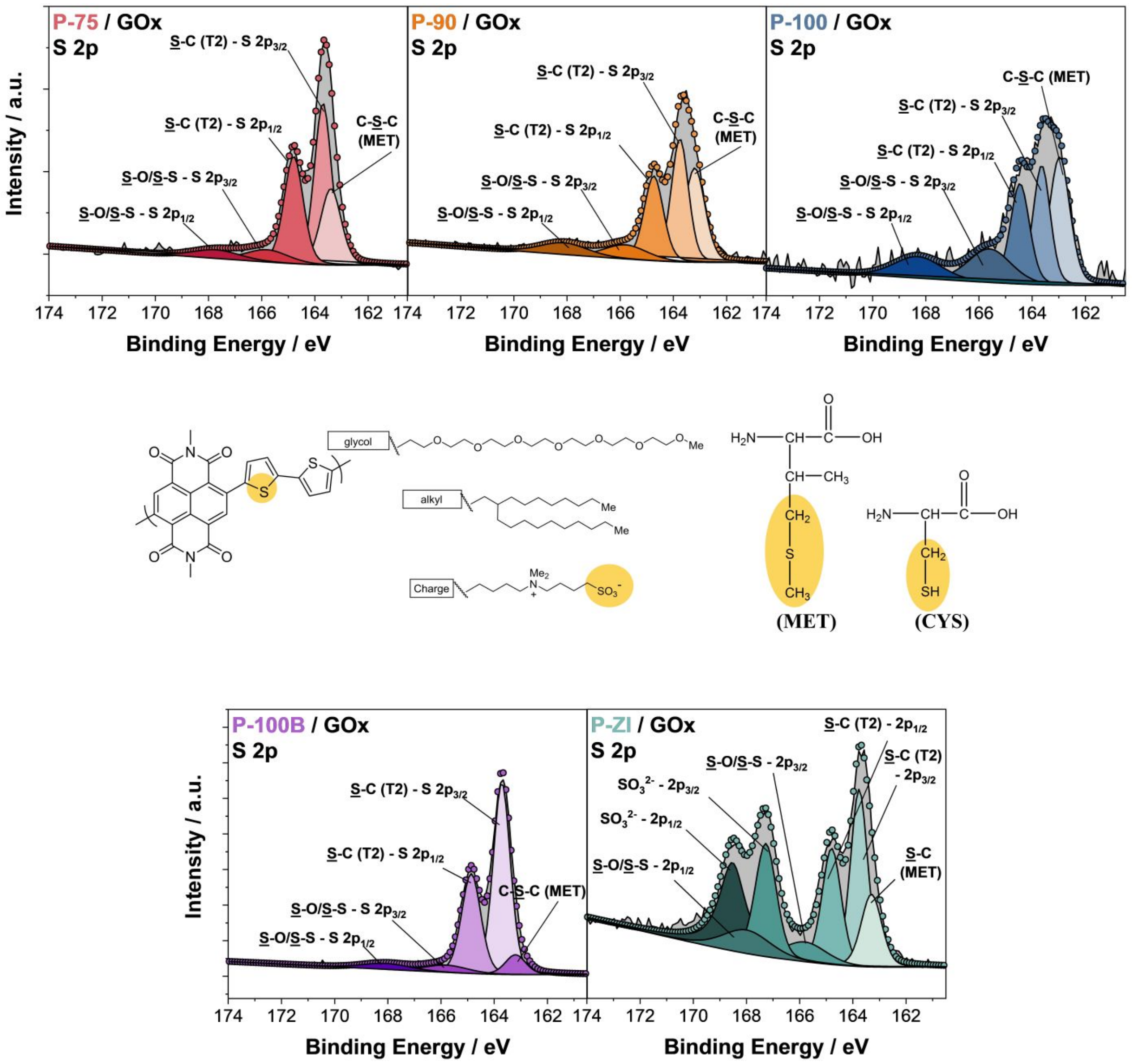

Figure S22. High-resolution of the S 2p XPS spectra of the polymer films after enzyme adsorption. Yellow circles highlight the chemical bonds containing sulfur (S) including the NDI backbone and side chains, and those in the amino acids present in GOx. The amino acids displayed in the figure correspond to only a selected few of the GOx sequence: methionine (MET) and cysteine (CYS).

We observe other deconvoluted peaks in the S 2p spectra after GOx adsorption (e.g., methionine –MET, and possibly cysteine –CYS, [amino acids]). In particular, we observe a peak at

approximately 165.5-165.8 eV, which is more prominent in P-100 than in the other polymers. Many chemical groups can lead to this peak, including cysteine amino acid,[5] and oxidized methionine sulfur[6] since this residue is more exposed to the outer environment (i.e. reactive oxygen species) due to the hypothesized expanded conformation of GOx on P-100. Nevertheless, given that P-100 generally retains the least amount of enzyme on its surface, stronger XPS signals suggest a higher concentration of the investigated species, which is only possible if more of this species is exposed to the XPS beam.

Table S7. Deconvolution of the S 2p XPS spectra is shown in **Figure S22**.

**Bonds of interest**		**P-75**	**P-90**	**P-100**	**P-100B**	**P-ZI**
**C-S-C (MET)**	**B.E. (eV)**	163.400	163.200	163.000	163.200	163.300
	**Area**	196.277	242.138	89.524	118.004	218.613
	**FWHM (eV)**	0.910				
**S-C (T2) – $2p_{3/2}$**	**B.E. (eV)**	163.69	163.76	163.64	163.72	163.76
	**Area**	347.384	264.422	69.659	955.707	445.503
	**FWHM (eV)**	0.760				
**S-C (T2) – $2p_{1/2}$**	**B.E. (eV)**	164.79	164.74	164.46	164.86	164.79
	**Area**	257.511	203.138	65.461	556.144	326.988
	**FWHM (eV)**	0.850				
**S-O/S-S – $2p_{3/2}$**	**B.E. (eV)**	165.800	165.800	164.600	165.800	165.800
	**Area**	65.524	74.957	48.601	89.556	122.971
	**FWHM (eV)**	1.890				
**S-O/S-S – $2p_{3/2}$**	**B.E. (eV)**	167.700	168.130	168.290	168.070	168.000
	**Area**	50.773	69.626	34.686	81.620	163.396
	**FWHM (eV)**	2.100				
**SO_3 - $2p_{3/2}$**	**B.E. (eV)**	-	-	-	-	167.27
	**Area**	-	-	-	-	351.833
	**FWHM (eV)**	0.940				
**SO_3 - $2p_{1/2}$**	**B.E. (eV)**	-	-	-	-	168.53
	**Area**	-	-	-	-	430.060
	**FWHM (eV)**	1.168				

B.E. = Binding energy
FWHM = Full width at half maximum

Table S8. Binding energy shifts of the C 1s peaks after enzyme adsorption. The C 1s spectra before and after enzyme adsorption are displayed in **Figures S20** and **S21**, respectively.

**Δ B.E. (eV)**	**C=C**	**C-C, C-H**	**C-S, C-N**	**C-O**	**C=O**
**P-75**	0.024	0.132	0.234	0.122	-0.118
**P-90**	-0.115	-0.018	0.09	0.135	-0.304
**P-100**	-0.081	-0.103	-0.006	0.409	-0.406
**P-100B**	-0.028	0.491	0.017	0.063	-0.102
**P-ZI**	0.092	0.098	0.121	0.125	-0.021

B.E. = Binding energy

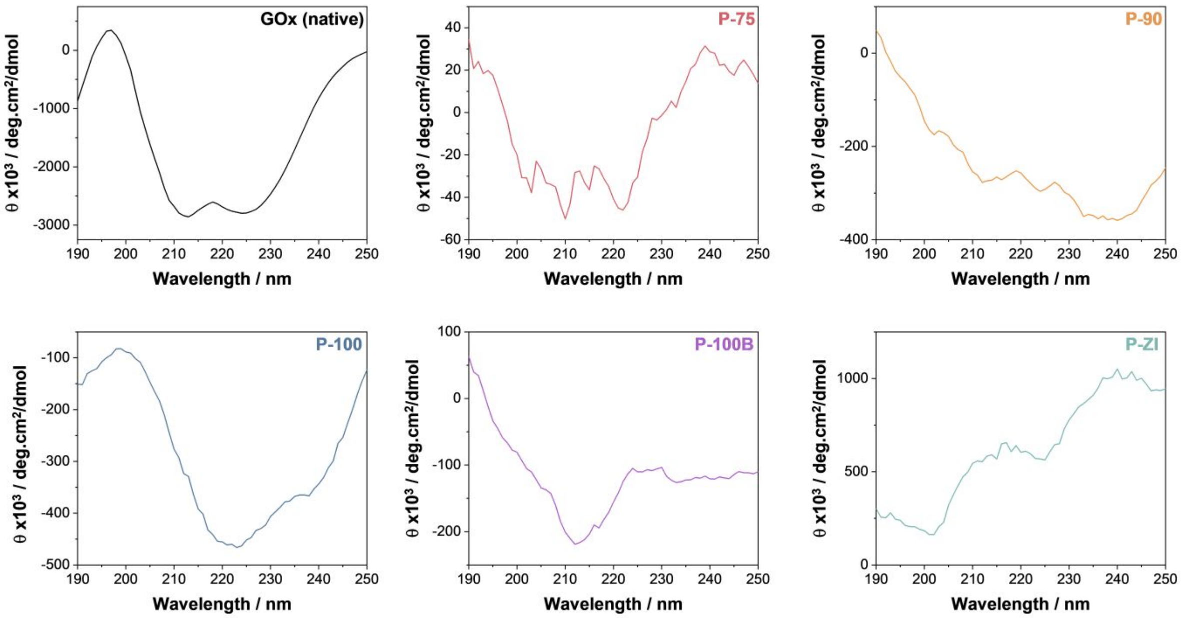

Figure S23. CD spectra of GOx in solution (native state) and adsorbed on polymer surfaces. Different structural elements contribute to the characteristic CD spectrum of GOx.[7] The negative bands at 222 nm and 208 nm and a positive band at 193 nm are general characteristics of α-helical conformation.[8] The β-sheets have negative bands at 218 nm and positive bands at 195 nm,[9] whereas very low ellipticity above 210 nm and negative bands near 195 nm correspond to disordered conformation.[10] Typically, the difference in absorption between right and left-polarized light in the far- and near-UV regions (190 to 250 and 250 to 300 nm, respectively) is recorded as a fingerprint of the biomolecule structure.

Supplementary Discussion 1. Effect of surface charge and wettability on protein adsorption. Proteins are thought to undergo translational and rotational motions to find a preferred orientation before adsorption, dictated by the properties of the underlying substrate surface.[11] The favored orientation of a protein on the surface is related to its free energy minimum, which depends on various surface-related parameters, such as Coulomb and Van der Waals interactions, hydrogen bonds, and entropy gain of solvent molecules or counter-ion release.[12] The substrate-protein interactions may lead to an increase in protein's free energy, ultimately maximizing its footprint through conformational reorganization.[12] Attwood et al. demonstrated through an empirical model that surface charge and hydrophobicity were the primary adsorption drivers, tested on various proteins on different self-assembled monolayers.[13]

Effect of surface charge. Xie et al. studied orientations of GOx on differently charged surfaces via molecular simulations.[11] The authors demonstrated that GOx adsorbed in different configurations depending on the underlying surface charge (**Figures 3** and **S9**). On positively charged surfaces, such as P-100B, the negatively charged GOx (isoelectric point –PI 4.2) can adsorb in a "standing" fashion, where the substrate-binding domain is accessible to glucose. However, as the surface charge density decreases, Van der Waals interactions become more prominent, and more possible orientations can occur where the protein ultimately preferentially adopts an unfavorable "front-lying" position where the substrate-binding domain is inaccessible, as assumed to be the case for P-ZI. On negatively charged surfaces (P-75, P-90, and P-100), although GOx is also overall negatively charged, it can still adsorb through its positively charged lysine or neutral residues (**Figures S9** and **Table S3**), adopting a "back-lying orientation" with the substrate-binding site facing outward and the redox-active center facing the polymer surface.[11]

In addition, the relatively high ionic strength of the medium (1X PBS) is expected to influence GOx adsorption due to charge screening by ions in the solution. In some cases, the screening effect from the electrolyte has been shown to weaken electrostatic interactions, leaving only Van der Waals interactions at play.[12] However, the screening effect is not expected to be beneficial to the adsorption of GOx on a negatively charged surface. This effect may contribute to the decrease in the adsorbed mass for the mixed alkyl/glycol series, where the least negatively charged surface has the highest adsorbed mass. For the zwitterionic surface (P-ZI), only the Van der Waals interactions are expected to occur, suggesting an unfavorable front-lying orientation of GOx on the polymer surface.

Effect of surface wettability. Protein adsorption is less favored on hydrophilic surfaces due to the energetic cost of surface dehydration, where a contact angle of 65° has been reported as the hydrophilicity threshold for adsorption.[14] However, the literature is inconsistent on this matter.[12, 14] For example, Anand et al. postulated that changing surface polarity affects the adsorbed protein amount (increasing as the surface becomes less polar) and that increased surface polarity destabilizes the proteins (unfolding) due to stronger protein-protein and protein-surface interactions.[15] The authors suggested that a less polar surface leads to a more rigid adsorbed layer. In the case of glycoproteins, such as GOx, the hydrophobic side chains are directed inward.[16] Protein adsorption on hydrophobic/apolar surfaces is expected to lead to substantial conformational changes.[16]

Supplementary Discussion 2. Δ*d* vs. Δ*f* analysis.

We investigate the viscoelastic properties of the GOx adsorbed layer by plotting Δ*d* as a function of Δ*f* (**Figures 4d**, **S16**, and **Table S1**). The Δ*d* vs. Δ*f* plots represent viscoelastic changes in the protein layers as a function of the adsorbed mass.[17] Three main factors generally accounted for changes in dissipation: dissipations located at the (i) protein-substrate interface and (ii) protein-liquid interface (including effects of a change in surface roughness), and (iii) within the protein layer (including effects of trapped liquid as the available space changes dynamically with the adsorption process).[17] The Δ*d* vs. Δ*f* plots contain a series of linear regions with their characteristic slopes, where a high value indicates the formation of a loose protein layer.[17]

Figure S16 shows that all polymers, except P-ZI, presented 3 linear regions in the adsorption process. Phase ① indicates a relatively rigid (low dissipation vs. frequency) attachment until a critical surface coverage is reached. Phase ② exhibits either the attachment of a viscoelastic stratum onto the first layer or loose (i.e., imperfectly coupled) binding of an additional rigid layer onto the first. The adsorption of this layer seems to be partially reversible, as illustrated in the rinsing process. Phase ③ displays a further increase in the frequency, with a slight increase in dissipation, related to film thickening and possibly the removal of water molecules between loosely bound adlayers. Increasing the EG content decreases the rigidity of the first layer adsorbed on the films. P-ZI presents a unique case, revealing a two-step adsorption process. In the P-ZI phase ② stabilization, we observe a slight decrease in the Δ*d* value with an increase in Δ*f*, indicating a thickening of the enzyme layer and the possible removal of water molecules from the loosely bound adlayers.

During the washing phase, a rapid decrease in Δ*d* was observed for all polymers, concomitant with a slight Δ*f* decrease, which most likely is due to the outer (i.e., film-solvent located) loosely adsorbed GOx molecules being removed by the buffer. Afterward, during the stabilization of the rinsing process, we observe the opposite behavior for P-75/P-90 and P-100/P-100B. P-75 and P-90 display a relatively constant Δ*d* value (or slightly decreasing) with a decrease in the Δ*f* value, suggesting the formation of a looser protein layer rather than a more rigid one. In contrast, P-100 and P-100B exhibit a slight increase in Δ*f* and Δ*d* values (more evident for P-100B) after stabilization, suggesting a configuration change in the existing bound layers, possibly the formation of a looser and more hydrated structure upon introducing a new buffer (especially for P-100B). On the other hand, the washing phase of P-ZI films shows a sharp decrease in Δ*d* with a slight decrease in the Δ*f* value, suggesting a significant stiffening of the layer. This is also indicated by the "negative" value of the dissipation upon rinsing (negative since we take the swollen polymer as the baseline). Upon stabilization following the washing step, an increase in Δ*d* occurred with a relatively constant Δ*f*, indicating a possible relaxation of the enzyme layer.

References

1. Moia, D.; Giovannitti, A.; Szumska, A. A.; Maria, I. P.; Rezasoltani, E.; Sachs, M.; Schnurr, M.; Barnes, P. R. F.; McCulloch, I.; Nelson, J., Design and Evaluation of Conjugated Polymers with Polar Side Chains as Electrode Materials for Electrochemical Energy Storage in Aqueous Electrolytes. *Energy Environ. Sci* **2019,** *12* (4), 1349-1357.
2. The UniProt Consortium, Uniprot: The Universal Protein Knowledgebase. *Nucleic Acids Research* **2016,** *45* (D1), D158-D169.
3. Frederick, K. R.; Tung, J.; Emerick, R. S.; Masiarz, F. R.; Chamberlain, S. H.; Vasavada, A.; Rosenberg, S.; Chakraborty, S.; Schopfer, L. M.; Schopter, L. M., Glucose Oxidase from Aspergillus Niger. Cloning, Gene Sequence, Secretion from Saccharomyces Cerevisiae and Kinetic Analysis of a Yeast-Derived Enzyme. *Journal of Biological Chemistry* **1990,** *265* (7), 3793-3802.
4. Gasteiger, E.; Hoogland, C.; Gattiker, A.; Duvaud, S. e.; Wilkins, M. R.; Appel, R. D.; Bairoch, A., Protein Identification and Analysis Tools on the Expasy Server. In *The Proteomics Protocols Handbook*, Walker, J. M., Ed. Humana Press: Totowa, NJ, **2005**; pp 571-607.
5. Zubavichus, Y.; Fuchs, O.; Weinhardt, L.; Heske, C.; Umbach, E.; Denlinger, J. D.; Grunze, M., Soft X-Ray-Induced Decomposition of Amino Acids: An Xps, Mass Spectrometry, and Nexafs Study. *Radiation Research* **2004,** *161* (3), 346-358, 13.
6. Weser, U. In *Redox Reactions of Sulphur-Containing Amino-Acid Residues in Proteins and Metalloproteins, an Xps Study*, Berlin, Heidelberg, Springer Berlin Heidelberg: Berlin, Heidelberg, **1985**; pp 145-160.
7. Greenfield, N. J., Using Circular Dichroism Spectra to Estimate Protein Secondary Structure. *Nature Protocols* **2006,** *1* (6), 2876-2890.
8. Holzwarth, G.; Doty, P., The Ultraviolet Circular Dichroism of Polypeptides1. *J. Am. Chem. Soc.* **1965,** *87* (2), 218-228.
9. Greenfield, N. J.; Fasman, G. D., Computed Circular Dichroism Spectra for the Evaluation of Protein Conformation. *Biochemistry* **1969,** *8* (10), 4108-4116.
10. Venyaminov, S. Y.; Baikalov, I. A.; Shen, Z. M.; Wu, C. S. C.; Yang, J. T., Circular Dichroic Analysis of Denatured Proteins: Inclusion of Denatured Proteins in the Reference Set. *Analytical Biochemistry* **1993,** *214* (1), 17-24.
11. Xie, Y.; Li, Z.; Zhou, J., Hamiltonian Replica Exchange Simulations of Glucose Oxidase Adsorption on Charged Surfaces. *Phys. Chem. Chem. Phys.* **2018,** *20* (21), 14587-14596.
12. Rabe, M.; Verdes, D.; Seeger, S., Understanding Protein Adsorption Phenomena at Solid Surfaces. *Advances in colloid and interface science* **2011,** *162* (1-2), 87-106.
13. Attwood, S. J.; Kershaw, R.; Uddin, S.; Bishop, S. M.; Welland, M. E., Understanding How Charge and Hydrophobicity Influence Globular Protein Adsorption to Alkanethiol and Material Surfaces. *J. Mater. Chem. B* **2019,** *7* (14), 2349-2361.
14. Vogler, E. A., Protein Adsorption in Three Dimensions. *Biomaterials* **2012,** *33* (5), 1201-1237.
15. Anand, G.; Sharma, S.; Dutta, A. K.; Kumar, S. K.; Belfort, G., Conformational Transitions of Adsorbed Proteins on Surfaces of Varying Polarity. *Langmuir* **2010,** *26* (13), 10803-10811.
16. Seehuber, A.; Dahint, R., Conformation and Activity of Glucose Oxidase on Homogeneously Coated and Nanostructured Surfaces. *The Journal of Physical Chemistry B* **2013,** *117* (23), 6980-6989.
17. Singh, K.; Blanford, C. F., Electrochemical Quartz Crystal Microbalance with Dissipation Monitoring: A Technique to Optimize Enzyme Use in Bioelectrocatalysis. *ChemCatChem* **2014,** *6* (4), 921-929.
